# Ionic Mechanisms of Propagated Repolarization in a One-Dimensional Strand of Human Ventricular Myocyte Model

**DOI:** 10.3390/ijms242015378

**Published:** 2023-10-19

**Authors:** Yukiko Himeno, Yixin Zhang, Suzuka Enomoto, Hiroto Nomura, Natsuki Yamamoto, Shotaro Kiyokawa, Mirei Ujihara, Yuttamol Muangkram, Akinori Noma, Akira Amano

**Affiliations:** Department of Bioinformatics, College of Life Sciences, Ritsumeikan University, Shiga 525-8577, Japan; sj0041xh@ed.ritsumei.ac.jp (Y.Z.); noma@sk.ritsumei.ac.jp (A.N.); a-amano@fc.ritsumei.ac.jp (A.A.)

**Keywords:** repolarization propagation, mathematical 1D strand model, human ventricular myocyte, early afterdepolarization, late sodium current

## Abstract

Although repolarization has been suggested to propagate in cardiac tissue both theoretically and experimentally, it has been challenging to estimate how and to what extent the propagation of repolarization contributes to relaxation because repolarization only occurs in the course of membrane excitation in normal hearts. We established a mathematical model of a 1D strand of 600 myocytes stabilized at an equilibrium potential near the plateau potential level by introducing a sustained component of the late sodium current (*I*_NaL_). By applying a hyperpolarizing stimulus to a small part of the strand, we succeeded in inducing repolarization which propagated along the strand at a velocity of 1~2 cm/s. The ionic mechanisms responsible for repolarization at the myocyte level, i.e., the deactivation of both the *I*_NaL_ and the L-type calcium current (*I*_CaL_), and the activation of the rapid component of delayed rectifier potassium current (*I*_Kr_) and the inward rectifier potassium channel (*I*_K1_), were found to be important for the propagation of repolarization in the myocyte strand. Using an analogy with progressive activation of the sodium current (*I*_Na_) in the propagation of excitation, regenerative activation of the predominant magnitude of *I*_K1_ makes the myocytes at the wave front start repolarization in succession through the electrical coupling via gap junction channels.

## 1. Introduction

It has long been recognized that the repolarization of cardiac action potential (AP) can be regulated by both intrinsic ion channel properties, as well as cable properties conferred by the three-dimensional syncytium of cardiac myocytes [1,2,3,4]. However, the mechanisms of the propagation of repolarization in multicellular preparations of the cardiac muscle remain to be clarified based on ionic current systems. Initially, Weidman (1951) [5] observed all-or-none repolarizations in sheep’s Purkinje fiber when he applied a hyperpolarizing current beyond a certain strength during the plateau phase. He observed that there was a definite ‘threshold’ for the all-or-none repolarization, and suggested that the wave of repolarization could propagate without decrement as in the case of excitation. Essentially the same threshold phenomenon was observed by Cranefield and Hoffman (1958) [6] when inducing repolarization in the papillary muscle preparation. However, attempts to determine the velocity of the propagation of repolarization were not entirely successful. In part, this were due to the fact that the velocity of the repolarization wave was much slower than that of the excitation, so the propagation of repolarization was expected to be overtaken by the normal process of repolarization after the beginning of the AP. 

The first theoretical study of cardiac electrophysiology using detailed mathematical descriptors was presented by Noble in 1962 [1]. He modified the Hodgkin–Huxley equations of squid giant axon and combined these with the cable theory to quite accurately reproduce many of the electrical properties of Purkinje fiber APs and pacemaker potentials. Purkinje fiber taken from the conduction system of juvenile sheep hearts had an exceptionally large diameter and length, which allowed extrapolation of the theory developed for the squid nerve to the cardiac muscle preparation after some modifications on parameters. After the epoch-making experimental milestones of the introduction of both dissociated cardiomyocyte preparations [7,8,9] and the patch clamp technique [10] in the next decades, the characteristics of ion channels and transporters were extensively clarified in the isolated cardiac myocytes from various cardiac tissues. These milestones made possible the development of various cardiac myocyte models based on the experimental data on isolated single myocytes [11]. Since then, cardiac myocyte models have become increasingly detailed and have been validated based on experimental data (for human ventricular myocyte models, ORd model [12], GPB model [13], Asakura et al., [14], Himeno et al. [15], etc.). 

The aims of the present simulation study were (1) to clarify the ionic mechanisms of induced regenerative repolarization and the threshold phenomenon for the all-or-none repolarization in dissociated ventricular myocytes, (2) to evaluate the impacts of intercellular currents on the propagation of repolarization induced in the myocyte strand, and (3) to determine the velocity of the propagation of the repolarization wave. Accordingly, we first determined whether our comprehensive human ventricular myocyte (hVC) model [15] could reproduce the all-or-none repolarization as demonstrated by Trenor et al. (2017) [2] using the ORd model [12]. After confirming the fundamental behavior of all-or-none repolarization in the hVC model, we developed an in silico 1D strand of myocytes to analyze the effects of the intercellular currents on the regenerative repolarization as well as on the propagation of the repolarization wave. The clinical implications for the antiarrhythmic treatment of the present study will be described in the Section 3. 

## 2. Results

### 2.1. Threshold Potentials for the All-or-None Repolarization in the hVC Model

**Figure 1:**
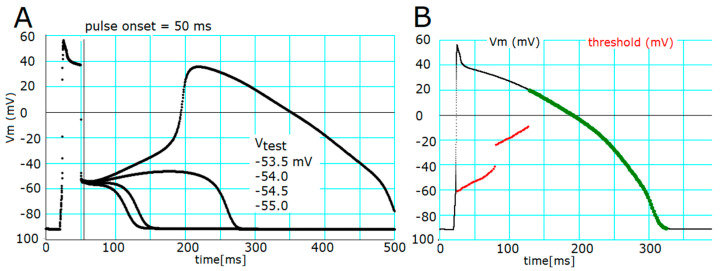
Threshold potentials detected by applying *V*_t_ of 5 ms in duration to different levels in the hVC model. (**A**) The application of *V*_t_ to −53.5, −54, −54.5, and −55 mV induced dynamic changes in the time course of plateau potential as displayed from top to bottom (four traces), respectively. The black vertical line in panel A indicates the off time (t = 55 ms) of the 5 ms clamp pulse. (**B**) The threshold potentials at which the given clamp pulse induced abolition of the plateau potential (red) were plotted against the off time of various test pulses. Green dots indicated there were no threshold potential. In this range colored in green, *V*_t_ did not induce all-or-none repolarization but led to monotonic repolarization.

Before examining the propagation of repolarization, we examined whether the all-or-none repolarization, i.e., the key experimental basis for suggesting the propagation of repolarization, can be observed in a single hVC model as has been proved in the ORd model by Trenor et al. (2017) [2]. In the simulation in Figure 1, the membrane potential (*V*_m_) was clamped at various test potential (*V*_t_) levels with 0.5 mV intervals for a duration of 5 ms. 

After the membrane was released from the temporal voltage clamp, *V*_m_ changed automatically before progressive depolarization or hyperpolarization became obvious as seen in Figure 1A. The slope of this change reversed between −55 and −53.5 mV of the clamp pulse applied at 50 ms. By adjusting the *V*_t_ bit-by-bit, a relatively flat phase of *V*_m_ change, reflecting an unstable equilibrium, was obtained at around −50 mV. Above that level, delayed activation of an early after-depolarization (EAD)-like event started after the release time. A *V*_t_ far more positive than this threshold potential level induced the rising foot of an EAD-like event directly, while a more negative *V*_t_ induced monotonic repolarization. The threshold potentials, determined at an interval of 2 ms during the plateau phase, were plotted in Figure 1B (red dots). The threshold potential was time- and *V*_m_-dependent; it shifted to more negative levels when the clamp pulse was applied earlier during the AP plateau. There were two apparent ‘jumps’ in the traces of the thresholds: at *V*_t_ = −42 and −23 mV. The green dots on the control time course of AP (black trace) indicated that the hyperpolarizing clamp pulse only accelerated the monotonic repolarization. All these findings were very similar to those reported in previous studies [2,3]. 

### 2.2. Ionic Mechanisms Responsible for Determining the Threshold Potentials for All-or-None Repolarization 

**Figure 2:**
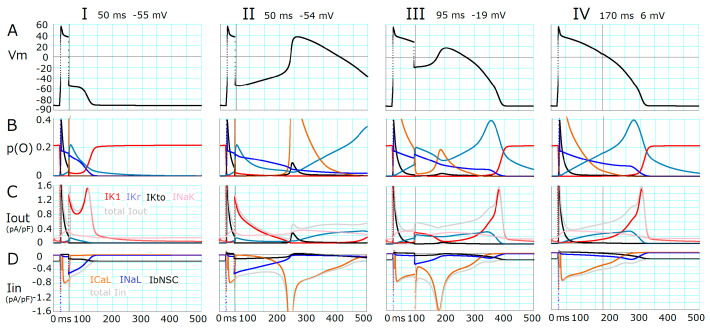
Ionic mechanisms underlying changes in *V*_m_ induced by applying 5 ms hyperpolarizing clamp pulses. Each time and voltage of the brief hyperpolarizing voltage clamp pulse applied were indicated at the top of each column. (**A**) indicates *V*_m_, (**B**) indicates the open probabilities *p*(O) of currents, *I*_K1_ (red), *I*_Kr_ (steel blue), *I*_Kto_ (black), *I*_CaL_ multiplied by 2 (chocolate), and *I*_NaL_ multiplied by 100 (blue), (**C**,**D**) indicate amplitudes of outward (*I*_out_) and inward (*I*_in_) currents, respectively. The same colors were used as in (**B**). The sum of major outward and inward currents was calculated and illustrated in gray. The amplitude of *I*_NaK_ (pink) and *I*_bNSC_ (black) are also shown in (**C**) and (**D**), respectively. The vertical lines indicate the end of the clamp pulse.

As demonstrated in Figure 1A, the fate of all-or-none repolarization was determined by the initial slow *V*_m_ changes after switching off the clamp pulse (Figure 1A). To clarify the ionic mechanisms underlying the initial slow change, the changes in *p*(O) were plotted in Figure 2B. In both columns I and II, hyperpolarizing clamp pulses were applied at 50 ms. When the pulse induced repolarization to −55 mV, the *p*(O) of inward-going late sodium current (*I*_NaL_), *p*(O)*_I_*_NaL_, gradually decreased, and *p*(O) of the rapid component of delayed rectifier potassium current (*I*_Kr_), *p*(O)*_I_*_Kr_, also decreased. The magnitude of *p*(O) of the inward rectifier potassium channel (*I*_K1_), *p*(O)*_I_*_K1_, was slightly but significantly increased to enlarge *I*_K1_ (Figure 2C) by the negative jump in *V*_m_ from the plateau potential to −55 mV. The sum of the K^+^ currents was larger, by about 0.3 pA/pF, than the sum of the major inward currents, which were mainly given by *I*_NaL_ and the background non-selective cation current, *I*_bNSC_, to drive the gradual repolarization (sum of major *I*_out_ and total *I*_in_ were represented by gray lines in Figure 2C and 2D, respectively). Finally, the membrane quickly hyperpolarized to the resting potential via a positive feedback cycle; the rapid increase in *p*(O)*_I_*_K1_ drives repolarization and more repolarization induces an increase in *p*(O)*_I_*_K1_. Through this cycle, the amplitude of *I*_K1_ showed a sharp peak as large as 1.5 pA/pF at about −70 mV. From this fact, it may be concluded that the *I*_K1_ takes the central role in inducing regenerative repolarization toward the resting potential.

**Figure 3:**
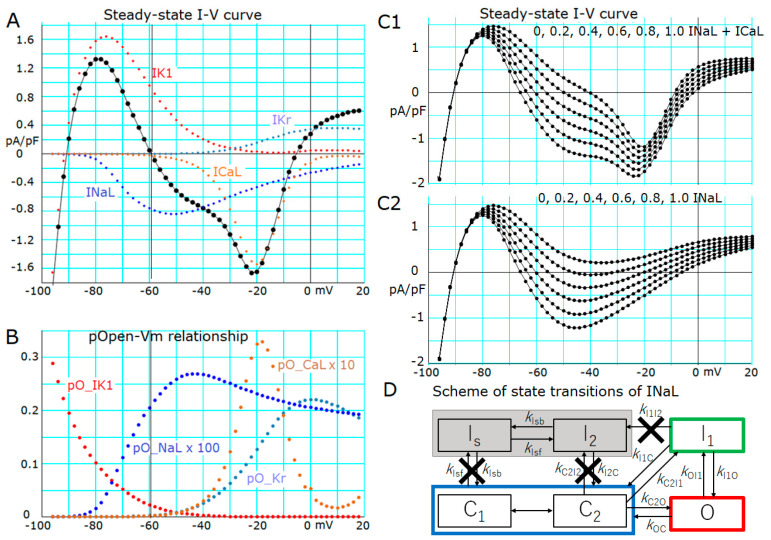
The N-shaped *I*-*V* curve with two stable equilibrium potentials and one unstable equilibrium potential of the hVC model. The *I*-*V* curves in (**A**,**C1**,**C2**) were measured by applying the voltage clamp test pulses of 4.9 s in duration given every 2 mV step from the holding potential of −80 mV. Current magnitudes saturated within the duration of test pulses at the end of individual test pulses, and were plotted in these figures. Additionally, (**A**) shows total whole cell current (black) and current components *I*_K1_, *I*_Kr_, *I*_NaL_, and *I*_CaL_ depicted in different colors as indicated. The steady-state *p*(O)s of major ion channels were plotted in (**B**). Note that *p*(O)*_I_*_NaL_ and *p*(O)_*I*CaL_ were plotted after multiplication by 100 and 10, respectively. (**C1**,**C2**) shows the influence of increasing the amplitude of *I*_NaL_ on the appearance of equilibrium points in the presence and the absence of *I*_CaL_, respectively. Increasing the amplitude of *I*_NaL_ shifted the N-shaped *I*-*V* curve negatively. All *I*-*V* and *p*(O)-*V* relationships share the same abscissa of *V*_m_. In (**D**), the state of slow inactivation, I_s_ and the transitional inactivated state, I_2_ of *I*_NaL,_ were shaded by gray color to indicate that these states were frozen, namely, the state transition from other states was totally prevented as indicated by cross marks. Thereby, repetitive state transitions between I_1_ and O produce continuous bursts of brief openings of the channel, and ‘late scattered mode’ of Na channel [16] (*k*_I1O_ is close to *k*_OI1_) during a clamp pulse to depolarized potentials. If integrated over bursts with numerous numbers of brief openings, the whole-cell *I*_NaL_ maintains a significant amplitude during depolarization. With an intact transition scheme, *I*_NaL_ gradually decreases in amplitude through the state transition from O to (I_s_ + I_2_) when the membrane is depolarized. The state transition between the two closed states (**C1**,**C2**) was assumed to be instantaneous on the *V*_m_ jump; the probability of (**C2**) increases with membrane depolarization. In the state transition of transient sodium current (*I*_NaT_) (see Supplementary Materials) the I_1_ state is absent, and the inactivation occurs through a state transition from O to I_2_ at transition rate *k*_OI2_, which has the same magnitude as *k*_OI1_.

When the hyperpolarization from the plateau potential was increased only by 1 mV (to −54 mV), the slope of the slow depolarization was inverted from a negative (at −55 mV) to a positive direction (column II). As expected, no significant changes were obvious in the initial levels of *p*(O) or the amplitudes of *I*_NaL_, *I*_Kr_, *I*_bNSC_, and *I*_K1_ immediately after the break in the voltage clamp pulse in comparison to the pulse to −54 mV. However, an evident exponential rise in the L-type calcium current (*I*_CaL_) amplitude was observed 200 ms after the pulse (Figure 3D); the more the *p*(O)*_I_*_CaL_ increased, the more depolarized the membrane became, and vice versa. The accompanying changes in *I*_NaL_, *I*_Kr_, *I*_bNSC_, and *I*_K1_ may occur secondarily due to the membrane depolarization caused by *I*_CaL_. The final discharge of an EAD-like event is caused by the much-enhanced positive feedback cycle of *I*_CaL_ activation. 

In column III, the *V*_t_ was −19 mV, which gave a stable *p*(O)*_I_*_CaL_ of about 0.02 to supply an *I*_CaL_ amplitude comparable to *I*_NaL_ at *V*_t_ = −54 mV (column II). On the other hand, the *I*_NaL_ amplitude was smaller if compared with that obtained by *V*_t_ at −54 mV because of *V*_m_-dependent inactivation and the smaller driving force for the carrier ion Na^+^. The *I*_CaL_ was also partially inactivated to give the decelerated rising phase of the EAD-like event. In column IV, the *V*_t_ (at 6 mV) was very close to the intrinsic plateau potential at 170 ms, and thereby the time courses of each current were almost the same as in the natural time course of final repolarization of the plateau. There was no threshold potential observed thereafter. It is important to note that the *I*_Kto_ is activated by the rapid depolarization, but is almost instantaneously deactivated by the hyperpolarization induced by the temporal voltage clamp pulse, thereby, *I*_Kto_ plays little role in initiating the full repolarization of the plateau (see Näbauer et al., 1996 [17]).

In summary, the *I*_K1_ takes the primary role in generating regenerative repolarization and the *I*_CaL_ plays a role in regenerative depolarization. Both *I*_NaL_ and *I*_CaL_ play modulatory roles in determining the threshold potential of repolarization.

### 2.3. Induction of a Stable Equilibrium Potential in the Current-Voltage Relationship of the hVC Model 

In principle, if there is a second stable equilibrium potential in a more positive potential range than a resting membrane potential, as well as a negative conductance region in the steady-state *I*-*V* curve of a single myocyte model, it would be possible to induce a wave of repolarization in a multicellular preparation of myocyte model as predicted in the previous studies [1,2,3,4]. It is expected that the propagation of repolarization might be simply visualized in a 1D strand of such myocyte models whose *V*_m_ is set to another stable equilibrium potential. In brief, graded hyperpolarizing stimulus, which is strong enough to induce an all-or-none repolarization, is applied, it is expected that the repolarization would propagate as a wave based on the ionic mechanisms revealed for the threshold phenomenon at the single myocyte level (Figure 2): progressive increase in *p*(O)*_I_*_K1_ supplemented by deactivation of *I*_NaL_ and *I*_CaL_ and activation of *I*_Kr_.

A study by Chandler and Meves (1970) [18] used sodium fluoride to generate a long-lasting plateau in the AP of squid axons and reproduced the prolonged plateau potential mathematically by modifying the inactivation parameter of the sodium current (*I*_Na_). In human ventricular myocytes, a slow inactivating sodium current component (*I*_NaL_) has been described [16]. This *I*_NaL_ component in the human ventricle inactivates with a sufficiently fast time course to shape APD normally. However, it has been reported that the rate of slow inactivation of *I*_NaL_ is further delayed in heart failure, or by modulation of phospholipid associated with cardiovascular disease, to prolong the AP duration [19]. Based on these findings, we removed the inactivation of *I*_NaL_ to obtain a stable equilibrium potential near the plateau potential [14]. Figure 3D shows the removal of the slow inactivation steps to states (I_s_ + I_2_), namely *k*_Isf_, *k*_Isb_, and *k*_I1I2_ were all set to zero. The fast inactivated state *I*_1_ allows flickering openings of the channel to reconstruct the repetitive brief openings demonstrated in the single channel recordings [16] because the magnitude of *k*_I1O_ was set close to *k*_OI1_.

The whole-cell current–voltage (*I*-*V*) relationships in Figure 3A were obtained by using the full set of current systems (Equation (3)) except for the slow inactivated states of *I*_NaL_ fixed at 0.45 for I_s_ and 0.001 for I_2_, respectively. The *I*-*V* curve represented by black points connected by lines clearly demonstrated the stable equilibrium (zero-current) potential at approximately −5 mV. Indeed, the slope of the whole-cell *I*-*V* curve was positive on both sides of the stable equilibrium potential. It should be noted that a similar positive slope was also evident around the stable equilibrium (resting) potential at −90.5 mV. In contrast, the slope of the steady-state *I*-*V* curve is negative around the unstable zero-current potential at −59 mV (vertical line).

To get deeper insights into the ionic mechanisms of the abolition of the AP plateau phase as well as the propagation of repolarization, we examined the composition of the whole-cell *I*-*V* relationship. In Figure 3A, the major determinants of *I*-*V* relations, such as *I*_K1_ (red), *I*_Kr_ (steel blue), *I*_NaL_ (blue), and *I*_CaL_ (chocolate) were superimposed on the whole-cell current. Toward the negative potential side of the threshold potential (vertical line), a sharp voltage-dependent increase in both *p*(O)*_I_*_K1_ and a decrease in *p*(O)*_I_*_NaL_ (blue dots in Figure 3B) were evident, indicating that resultant increase in outward *I*_K1_ and decrease in inward *I*_NaL_ were mainly responsible for the acceleration of repolarization. On the positive side of the threshold potential, the sharp *V*_m_-dependent increase in *p*(O)*_I_*_CaL_ and the resultant increase in *I*_CaL_ amplitude with depolarization promotes the depolarization. This influence of *I*_CaL_ activation was antagonized by the voltage-dependent increase in *p*(O)*_I_*_Kr_ to give the stable equilibrium potential at about −5 mV.

The *I*_NaL_ took a key role in inducing the second stable equilibrium potential at the voltage range of AP plateau as demonstrated in Figure 3C1,C2, where the amplitude of *I*_NaL_ was decreased from one to zero in steps of 0.2. In Figure 3C1, it is evident that the unstable equilibrium potential was largely shifted toward negative potentials from −40 mV to −60 mV with increasing magnitude of *I*_NaL_. If the limiting conductance of *I*_K1_ was increased or decreased by +20%, the unstable equilibrium potential was shifted only by +2 mV or −3 mV, respectively (not shown). In Figure 3C2, the ‘window component’ of *I*_CaL_ was removed to reveal the role of *I*_NaL_ in inducing the unstable equilibrium potential. In the absence of *I*_CaL_, two intersections of the *I*-*V* relation with the zero-current level disappeared when *I*_NaL_ was intact, shown in in Figure 3C2, leaving the stable equilibrium potential at the resting potential intact. The addition of *I*_CaL_ scarcely affected the location of unstable equilibrium potential when the relative magnitude of *I*_NaL_ remained in the physiological range (>0.2). If the gating kinetics of *I*_NaL_ (illustrated in Figure 3D) are intact under the physiological condition, the time-dependent slow inactivation accumulates the *p*(I_s_)*_I_*_NaL_ to promote membrane repolarization to the resting potential. 

### 2.4. Propagation of Repolarization from the Second Stable Equilibrium Potential at Around 0 mV

**Figure 4:**
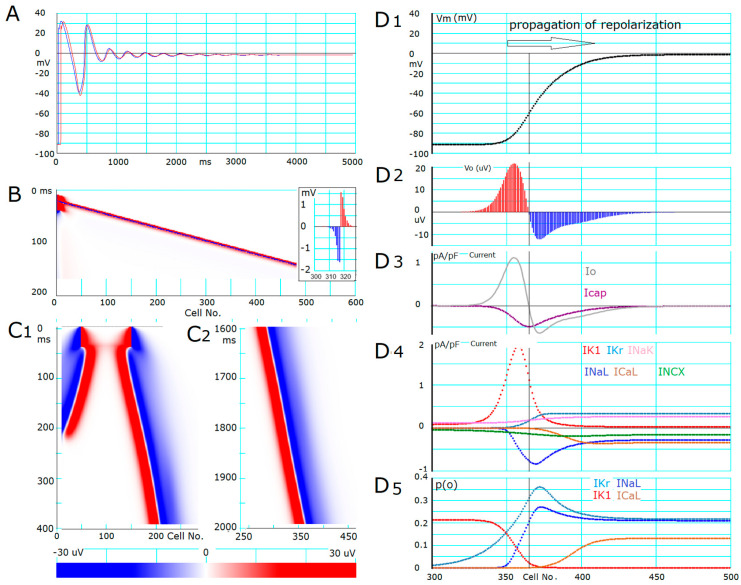
Propagated repolarization in a linear strand of the EAD-prone myocytes in comparison to that of excitation. Panel A shows the time course of *V*_m_, moving from the resting membrane potential (−91 mV) to the second stable equilibrium potential (−2 mV) in the myocyte strand, in which the deeply inactivated states (I_s_ + I_2_) of *I*_NaL_ were removed. Panel (**A**) indicates *V*_m_ recordings in cell No. 1 (blue) and 17 (red). The AP was evoked by applying a depolarizing pulse to cell No. 1 in the 1D strand of the hVC model. The *V*_m_ stabilized at about −3 mV at the end of the record in all myocytes within the strand. Panel (**B**) indicates the propagation of the O/I pattern (propagation of excitation) along the myocyte strand. The inset shows the stable O/I pattern of *V*_o_; positive *V*_o_ evoked by outward whole-cell current, *I*_o_ (red), or negative *V*_o_ due to inward *I*_o_ (blue). Note, that the cell number on the abscissa of the inset is the same as in panel B, but the scale of the *V*_o_ is reduced by 100 times. Panels (**B**,**C**) indicate the movement of O/I patterns (propagation of repolarization) along the myocyte strand by using the color code of *V*_o_ shown at the bottom of panel (**C**). The O/I patterns illustrated in (**C1**,**C2**) were obtained at different recording times. See text for the explanation of panels (**D1**–**D5**). Note that *p*(O)*_I_*_NaL_ and *p*(O)*_I_*_CaL_ were plotted after multiplication by 100 and 10, respectively, in panel (**D5**). The time scale shown in (**D5**) is common for all (**D1**–**D5**). The arrow head in (**D1**) indicates the direction of the propagation of repolarization. The scaling factor of *I*_K1_ = 1.2 was used in this simulation. Note that the scale of the *V*_o_ is reduced by 100 times in panel (**C**) if compared to that in panel (**B**).

To separate the propagation of repolarization from the natural time course of AP repolarization, the *V*_m_ of the myocyte strand was stabilized to the equilibrium potential near the plateau potential by fixing 45.1% of *I*_NaL_ channels to the inactivated states (I_s_ and I_2_). The stimulus current pulse (−45 pA/pF, 30 ms in duration) was applied to cell No. 1 (Figure 4A) at 10 ms after starting the *V*_m_ recording. The first AP of cell No. 1 (blue trace) was conducted sequentially from myocyte to myocyte in the 1D strand model of hVCs (the AP depicted in red is recorded from cell No. 17). With the modified *I*_NaL_ (shown in Figure 3A,D), a train of EADs was initiated spontaneously during the repolarizing phase of the AP, but the EAD subsequently transformed into decremental oscillations in the myocyte strand to level off at a stable depolarization (Figure 4A) as predicted by the steady-state *I*-*V* curve.

The rate of AP conduction was measured from the slope of the profile of *V*_o_ along the 1D strand of in silico myocytes (Figure 4B). An example snapshot of the original *V*_o_ profile along cell No. on the *x* axis from cell No. 300~330 is demonstrated in the inset of Figure 4B. The *V*_o_ of a given myocyte within the myocyte strand is positive (here depicted in red) when the whole-cell membrane current is outward and vice versa (depicted in blue). It was evident that the direction of *I*_o_, which corresponded to the sign of *V*_o_, was inverted from negative to positive between cell No. 317 and 318. The wavefront of excitation propagation (in the right-ward direction) was defined by the location of this inversion of the sign of the *I*_o_ (defined as O/I center). In Figure 4B, the *V*_o_ of an individual myocyte was represented by the color code indicated at the bottom of Figure 4C, in which the ordinate is time, and the abscissa represents cell No. of the myocyte strand. The propagation rate of membrane excitation was determined to be 48.0 cm/s from the slope of the linear relationship between the propagation time and the number of the myocytes, whose length was 120 mm in the hVC model (Figure 4B). 

The propagation of repolarization could be evoked in the 1D strand model with 600 hVCs which was depolarized continuously, as in Figure 4A. If compared with the propagation of excitation in panel B, the propagation of repolarization was characterized by a much smaller current density (by two orders of magnitude) of the major repolarizing current *I*_K1_ than that of the major excitatory current *I*_Na_. To trigger the propagation of repolarization in the myocyte strand, the hyperpolarizing current should be applied either to a large fraction of myocytes within the strand, or at a large amplitude, or for a longer duration of current pulse to overcome the buffering action of the rest fraction of myocytes in the strand. This was indicated by the findings demonstrated in Figure 4C1, where a trigger current pulse of 400 pA/pF and 40 ms in duration was applied simultaneously to 100 myocytes (cell No. 50~150). Immediately after switching off the test pulse, the distance between the two O/I centers on the left and right sides at the borders of stimulated and unstimulated myocytes slightly decreased for ~10 ms before starting the propagation of repolarization. If the triggering pulse was smaller (<310 pA/pF), and/or shorter in duration, or applied to a smaller number of myocytes, the distance between the two O/I centers was shortened to push back the repolarization, which is consistent with the presence of a threshold phenomenon observed in the multicellular preparations. 

The repolarizing electric stimulus applied to the 1D strand model of myocytes could be replaced by increasing the *G*_K1_, for example. Increasing the *p*(O)*_I_*_K1_ beyond 0.03 for 40 ms in the central 100-myocyte segment successfully triggered the propagation of repolarization. Similarly, the propagation of repolarization could be evoked by activation of the ATP-sensitive K^+^ channel (*I*_KATP_), which has a large maximum conductance (*G*_KATP_). Since the maximum conductance of *I*_Kr_ (*G*_Kr_) is much smaller than that of *I*_K1_, increasing the *p*(O)*_I_*_Kr_ failed to evoke hyperpolarization to trigger the propagation of repolarization. Hyperpolarizing stimulus applied by nullifying the *p*(O) of the major inward currents, *I*_NaL_ and *I*_CaL_, during the AP plateau phase, was not effective in inducing propagation of repolarization.

The rate of the propagation of repolarization (the slope of the movement of the O/I center) slightly decreased with time and reached a steady rate of 1.7 cm/s after ~400 ms. The ionic mechanisms of repolarization propagation were analyzed in panels D1–D5 in Figure 4, which shows snapshots taken 800 ms after the initiation of the triggering pulse. Surprisingly, the *V*_o_ profiles in Figure 4D2 were distributed over a much wider range of ~100 myocytes, in which *V*_m_ continues to repolarize (Figure 4D1), in contrast to the narrower width of *V*_o_ profile over 10 myocytes in the case of propagation of membrane excitation (inset in Figure 4B). The location of the O/I center is indicated by the vertical line in Figure 4D1–D5. On the left side of the O/I center, the *p*(O)*_I_*_K1_ increased and *p*(O)*_I_*_NaL_ decreased with a steep slope (Figure 4D5), and the amplitude of *I*_K1_ increased while the amplitude of *I*_NaL_ decreased (Figure 4D4). The profile of all variables shown in panels D1–D5 remained the same as far as the rate of propagation was constant, indicating that the propagation proceeded without decrement.

To compare the contribution of membrane ionic currents (*I*_m_) and extracellular current (*I*_o_) to varying *V*_m_ of individual myocytes during the propagation of repolarization, profiles of all related parameters are illustrated on a common *x*-axis of cell No. in the strand.

(1) $$I_{c}=I_{o}-I_{m}$$

where *I*_c_ (or *I*_cap_ in Figure 4D3) is the current through the membrane capacitor to determine d*V*_m_/dt, and *I*_m_ represents the sum of whole-cell ionic currents. Major ionic currents, such as *I*_K1_, *I*_Kr_, *I*_NaL_, and *I*_CaL_, and transporter currents *I*_NaK_ and I_NCX_ are depicted in different colors in Figure 4D4. It is evident that the peak amplitudes of these major currents are roughly in the same order of magnitude, indicating that each one of these major currents has the potential to affect the rate of propagation of+ repolarization. However, if the current amplitudes in Figure 4D4 were compared to the *V*_o_ or *I*_o_ profiles in Figure 4D2 or Figure 4D3, it would seem to indicate that *I*_K1_ and *I*_NaL_ took the central role in driving repolarization over a potential range more negative than −60 mV. On the right side of the O/I center, the increase in *p*(O)*_I_*_Kr_ and decrease in *p*(O)*_I_*_CaL_ over the segment of myocytes from No. 365 to 420 were mainly responsible for the initial repolarization over the *V*_m_ range from 0 to −60 mV. The currents generated by the Na/K pump (pink) and Na/Ca exchange (green) were relatively small and were mainly determined by the *V*_m_ change. 

It is of note that the large increasing outward current component of *I*_K1_ and decreasing *I*_NaL_ in the myocytes on the left side of the O/I center with *V*_m_ below −60 mV (red and blue in Figure 4D4) not only contribute to repolarize themselves but also repolarize the myocytes on the right side of the O/I center (see *I*_cap_ in Figure 4D3) through gap junction channels (see *I*_o_ in Figure 4D3). The area of the positive and negative *I*_o_ in Figure 4D3 was always equal and was distributed electrotonically along the strand. This electrotonic current started to repolarize the myocytes at the propagation wavefront where the inward and outward currents were still almost balanced at the potential near the quasi-equilibrium around 0 mV. These findings on current dynamics observed near the unstable equilibrium point around −60 mV were quite comparable, in qualitative aspects, with the ionic mechanisms observed in both the steady-state *I*-*V* curve (Figure 3) and the abolition experiment of the AP plateau (Figure 1 and Figure 2).

### 2.5. Effects of Varying the Conductance of I_NaL_, I_K1_, and Gap Junction Channel (G_g_) on the Rate of Propagation of Repolarization

The velocities of propagation for the repolarization were measured at various conductances of *I*_NaL_ and *I*_K1_ as listed in Table 1. The limiting conductance of *I*_NaL_ (*G*_NaL_) was increased by decreasing the magnitude of fixed (*p*(I_2_) *+ p*(I_s_)), and vice versa when decreasing *G*_NaL_. The rate of propagation of repolarization increased with decreasing *G*_NaL_, suggesting that the influence of *I*_NaL_ to interfere with the hyperpolarizing influence of *I*_o_ (= *I*_cap_ + *I*_m_) in myocytes located in front of the O/I center is greater than the accelerating influence of *I*_NaL_ deactivation in myocytes located behind the O/I center. As expected, an increase in the conductance of outward *I*_K1_ (*G*_K1_) increased the propagation of repolarization to support the central role of *I*_K1_ in promoting the propagation. 

In Table 1, the letter ‘F’ indicates that the triggering hyperpolarizing pulse failed to evoke the propagation of repolarization because of smaller *G*_K1_ and/or larger *G*_NaL_. Under these conditions, EAD-like events occurred in myocytes located before the wavefront after switching off the hyperpolarizing pulse. Then, the EAD-like event subsequently triggered an AP in myocytes behind the wavefront that stayed at the resting potential, resulting in a retrograde propagation of AP in the 1D strand. 

These findings, obtained by varying the gap junction conductance (*G*_g_), are consistent with the theory that the magnitude of the intercellular current is one of the determinants for promoting the rate of AP propagation (Table 2). 

## 3. Discussion

### 3.1. Brief Summary of the Results

(1) The ionic mechanisms underlying the induced all-or-none repolarization at the myocyte level (Figure 2), and the propagation of repolarization in a 1D linear strand of myocytes (Figure 4), were examined. Unambiguous propagation of repolarization that did not overlap with the natural time course of the AP plateau was observed only when the 1D strand of myocytes was depolarized at the second stable equilibrium potential, which was introduced by freezing the fraction of slow inactivation of *I*_NaL_ to 0.451 in Figure 4.
(2) The key ionic currents were *I*_K1_, *I*_Kr_, *I*_NaL_, and *I*_CaL_ in both the abolition of the AP plateau in a single ventricular myocyte and the propagation of repolarization in the in silico 1D strand of the myocyte model. Since the voltage range of the kinetic dynamics for *I*_K1_ and *I*_NaL_ are more negative compared to that for *I*_Kr_ and *I*_CaL_, as demonstrated in Figure 3A, *I*_K1_ and *I*_NaL_ play their role during the late phase of repolarization, while *I*_Kr_ and *I*_CaL_ work in the early phase of repolarization. The positive feedback mechanisms accelerate the rate of *V*_m_ change caused by the kinetics dynamics of these currents.
(3) In the abolition experiments in Figure 1 and Figure 2, the triggering pulse of hyperpolarization was supplied by the external current source, while the propagation of repolarization in the 1D strand was initiated by local current *I*_o_ and evoked by the myocytes located behind the wavefront of repolarization before the O/I center (Figure 4D2).
(4) The kinetic mechanisms of the four currents were most probably responsible for the stable propagation of repolarization.
(5) The O/I profile of *V*_o_ around the O/I center (Figure 4D2) is a mirror image of that in the propagation of excitation (Figure 4B, inset). The width of the O/I profile was much more elongated in the propagation of repolarization (Figure 4D1–D5) compared with that for the propagation of AP (Figure 4B), reflecting the relatively small current amplitude of *I*_K1_, *I*_NaL_, *I*_Kr_, and *I*_CaL_ in repolarization. The merit of using multiple channel species may be attributed to its flexibility in adjusting the configuration of the AP plateau phase more precisely and stably.
(6) The rate of propagation of repolarization was dependent mainly on the amplitude of *I*_K1_ and *I*_NaL_ (Table 1). The rate increased with increasing the *G*_g_ within a limited range of *G*_g_.

### 3.2. Clinical Implications

Noble et al. [1,3] demonstrated, for the first time, the principal ionic mechanisms underlying the induction of repolarization as well as the propagation of repolarization in cardiac myocytes, based on the computer model of cardiac membrane excitation. The present study used one of the detailed contemporary computer models (human ventricular myocyte models [12,13,14,15]) to clarify the ionic mechanisms of the propagation of repolarization in terms of individual ion channels as well as ion transporters to reconstruct the key experimental findings. The model was modified only by removing the very slow inactivation of *I*_NaL_, leaving other fast mechanisms of *I*_NaT_, *I*_CaL_, *I*_Kr_, the transient outward potassium current (*I*_Kto_), the slow component of delayed rectifier potassium current (*I*_Ks_), and *I*_K1_ intact to create the stable equilibrium potential near 0 mV. Since the kinetic time constant of the slow inactivation of *I*_NaL_ is in the order of several hundreds ms, fixing the *I*_NaL_ slow inactivation hardly interferes with the repolarizing process from the stable equilibrium potential. Thus, the ionic mechanisms of the repolarization demonstrated in the present study are all relevant to repolarization mechanisms under physiological conditions.

The ionic mechanisms underlying the propagation of repolarization might be examined using an in silico model of a simple 1D strand, in which hVCs were connected by gap junctions. Here, let us assume that the 1D strand is depolarized by preventing its repolarization by removing the slow inactivation step from the state transition scheme of *I*_NaL_ (Figure 3D). This strand will be connected to an intact 1D strand of a similar size. At the junction of the two strands, the *I*_K1_ channel in the latter strand will provide a hyperpolarizing current through gap junctions to the depolarized myocytes to repolarize toward the resting potential. This repolarization through the cell-to-cell interaction should propagate into the depolarized strand as demonstrated in Figure 4. Through this propagation of repolarization, the whole strand will recover to its resting potential. Thus, the propagation of repolarization might be relevant in clinical anti-arrhythmic treatment. Indeed, the present study found that the propagation of repolarization could be initiated by amplifying *G*_K1_ above 0.03, which is at the same level of *G*_K1_ at the resting potential, in a fraction of the in silico 1D strand of hVC model. As expected, it was found in our preliminary study that magnifying *G*_KATP_ was also useful in initiating the propagation of repolarization.

For the relevance of the present study on cardiac AP repolarization as well as the influences of these theoretical studies on clinical sciences, see the excellent review by Trenor et al. (2017) [2].

### 3.3. Ionic Mechanisms of Propagation of Repolarization in Comparison to the Propagation of Excitation 

Here, a comprehensive view of the propagation of repolarization is discussed in comparison to that of excitation, including the threshold phenomena for the abolition of the AP plateau phase, the steady-state *I*-*V* relations, and positive feedback mechanisms. The mechanisms of the propagation of membrane excitation in the cardiac tissue were well established, as described in many textbooks. In essence, the depolarization-dependent activation of *I*_Na_ and the *I*_Na_-dependent membrane depolarization cause the positive feedback cycle to promote the rapidly rising phase of AP. Depolarization of a myocyte within the 1D strand generates outward-going *I*_o_ in neighbor myocytes through gap junctions to cause the positive cycle of *I*_Na_ activation in the next myocyte. In contrast, the propagation of membrane repolarization takes place through the positive feedback cycle of repolarization-dependent activation of *I*_K1_ and *I*_K1_-dependent repolarization in the presence of supplemental ionic currents. Repolarization of myocytes in the strand induces inward-going *I*_o_ to repolarize myocytes which are still depolarized at the repolarization wavefront of the strand through gap junctions. 

In the intact hVC model, all the parameters of *I*_NaL_, including the slow inactivation states (I_2_ and I_s_) of the model, continuously change throughout the time course of the AP plateau phase driven by the time- and voltage-dependent gating parameters to promote the membrane repolarization. On the other hand, the state transitions among the C, O, and I_1_ states of *I*_NaL_ are much faster than the *V*_m_ change during the relatively slow AP plateau, and thereby these fast parameters roughly follow the steady-state values during the time course of the AP plateau phase. Thus, it is quite possible that the model may take nearly the same parameter set as that used in the steady-state *I*-*V* curve (Figure 3A) at an instant during the natural time course of the plateau phase. 

It seems difficult to find any ionic current that can substitute the role of *I*_NaL_ in the simulation of propagated repolarization in the multicellular preparations. Especially as the unstable equilibrium (threshold) potential at around −60 mV allowed the sharp inversion of *I*_o_ from inward to outward current at the O/I center, as demonstrated in Figure 4D1–D5. Note that these equilibrium potentials are common for both excitation and repolarization under physiological conditions (Figure 3). The window current of *I*_CaL_, which was depicted in chocolate in Figure 3A, can also provide the unstable equilibrium potential at about −40 mV in addition to the inducible stable equilibrium potential at −8.5 mV, even in the absence of *I*_NaL_ (Figure 3C1). However, around this threshold potential (−40 mV), the amplitude of the outward current (*I*_K1_) is much smaller than that at −60 mV, so the rate of repolarization may be too slow to evoke the regenerative propagation of repolarization against the buffering effect of the multicellular strand. In contrast, the threshold potential at ~−60 mV allowed the smooth and swift transition from the initial half of repolarization (ahead of the O/I center) to the final (behind the O/I center) repolarization due to the massive magnification of *I*_K1_ as shown in Figure 4D1–D5. The rapid upstroke of AP also starts at about −60 mV when the AP is triggered from the resting potential. 

Qu et al. [20] in their review paper, introduced a general framework to explain the mechanisms of EADs using the quasi-stable *I*-*V* relationships. The same framework is also useful in understanding how the second stable equilibrium potential was introduced in the present study. The description of a given current, *I*_X_, is described using three parameters; the limiting conductance *G*_X_, the open probability of the channel (*p*(O)_X_), and the driving force (*V*_m_ − *E*_X_), where *E*_X_ is the reversal potential of the ionic species of the current X.

(2) $$I_{X}=G_{X}\cdot p\left(O\right)\cdot \left(V_{m}-E_{X}\right)$$

The steady-state *I*-*V* curve is measured in experiments (or is computed in simulation in Figure 4) by clamping *V*_m_ until the *I*_m_ reaches a stable level at different voltages along the *V*_m_ axis. While the quasi-steady-state *I*-*V* curve in the present study was obtained by the same protocol, the magnitude *p*(O) of a selected slow parameter was fixed during the natural time course of the AP plateau; for example, the *p*(O)*_I_*_NaL_ in the present study (Figure 4C). Thus, at a depolarized *V*_m_ during AP plateau, the quasi-steady-state whole-cell *I*-*V* relationship shown in Figure 4C moves upward smoothly with the continuous reduction of *p*(O)*_I_*_NaL_ accompanied by increasing inactivated fraction (*p*(I_s_)*_I_*_NaL_ + *p*(I_2_)*_I_*_NaL_). At the plateau potential of the AP, the *V*_m_ traces the negative shift of the second stable equilibrium potential. Finally, the quasi-steady-state *I*-*V* relation fails to cross the X-axis over the depolarized potential range, and the membrane quickly repolarizes to the resting potential. Thus, the stable equilibrium potential could be induced in the 1D myocyte strand when the decrease of the inactivated fraction of *I*_NaL_ was frozen at an optimum level during the progress of the AP plateau with all the other kinetic parameters left intact. 

Lastly, some of the reported values of the velocity of repolarization in previous studies are compared. In the classic study of Cranefield and Hoffmann [6], using papillary muscle fiber, they stated, “no value was given for conduction velocity under Results because of the uncertainty of measurement” and described a velocity of 20 cm/s estimated from the records of a propagated repolarization wave in their Discussion. In addition, they commented on the value as “this value may not be correct, but it is certain that the velocity is low compared with that of propagated depolarization” and referred to the original study of Weidemann [5]. Our simulation using a strand composed of intact hVC demonstrated that it was impossible to determine a unique value for the velocity of propagated repolarization because the velocity increased continuously and approached infinity as the repolarization propagated [21]. If the slope of the O/I center was roughly calculated to be t = 205~255 ms after the hyperpolarizing stimulation, a value of 17 cm/s was obtained, which was in the same order of magnitude as that reported by Cranefield and Hoffmann. However, it should be noted that this velocity is not a pure rate of the propagation of repolarization, instead it is a rate of the propagation of repolarization overlapping the intrinsic spontaneous repolarization of each myocyte. Previous work in this area is of interest. There has been a study on the conduction across the interface between two different cell groups using micropatterned cocultures of genetically engineered (GE) excitable cells and neonatal rat ventricular myocytes (NRVMs) [22]. The GE cell was a monoclonal HEK293 cell line stably expressing human *I*_Na_ (Na_V_ 1.5), *I*_K1_ (Kir2.1), and rat connexin-43 (Cx43) gap junctions and had shorter APD (31.9 ms) than NRVMs (153.2 ms). They stimulated the co-cultured strand from the GE cell side and mapped the conduction of activation and repolarization using voltage-sensitive dye optically. In this strand, intrinsic repolarization occurred spontaneously in individual cells, but the GE cells of the former half triggered repolarization of the NRVMs composing the latter half of the strand because the APD of the GE cells was much shorter than NRVMs. If we calculate the rate of propagation of repolarization focusing on the donor–host interface from their result, the rate was about 1.8 cm/s which was much slower than that of activation (22 cm/s). In addition, Sperelakis et al. [23] measured the propagation of repolarization by inducing repolarization from another stable potential level to a resting potential as we did in the present study. They use a five-cell array of an extremely simplified model of electrical activity (2005). They reported that the rate of repolarization propagation is considerably (2.3~15 times) slower than that of excitation propagation, and that it varies with the number of gap junction channels (50~200 cm/s for repolarization propagation compared to 115~3000 cm/s for excitation propagation in the strand with 100~10,000 gap junction channels of 100 pS). Although the values previously reported in various experimental settings varied widely, the conclusion that the velocity of propagation of repolarization was much smaller than that of excitation was consistent in the present study. 

## 4. Materials and Methods

### 4.1. The hVC Model

A single ventricular myocyte having a cuboid shape (120 µm on the longitudinal axis, 37.6 µm on the transverse axis, and 8.4 µm in depth) was assumed to calculate the propagation rate of excitation and repolarization. The membrane ionic current (*I*_m_) was given as a sum of 15 components as described in Equation (3).

(3) $$I_{m}=I_{\mathrm{NaT}}+I_{\mathrm{NaL}}+I_{\mathrm{CaL}}+I_{\mathrm{Kto}}+I_{\mathrm{Kr}}+I_{\mathrm{Ks}}+I_{K1}+I_{\mathrm{Kpl}}+I_{\mathrm{bNSC}}+I_{\mathrm{Cab}}+I_{L\left(\mathrm{Ca}\right)}+I_{\mathrm{KATP}}+I_{\mathrm{NaK}}+I_{\mathrm{NaCa}}+I_{\mathrm{PMCA}}$$

As will be described in the present study, the *I*_K1_ takes a pivotal role in both the repolarization of the AP plateau as well as in the propagation of repolarization. In this simulation study, we used the *I*_K1_ model developed by the Ishihara group [24,25]. Fabbri et al. (2019) [26] compared six computer formulations of *I*_K1_ to overcome the deficiency of *I*_K1_ conductance in the human-induced pluripotent stem cells (hiPSC-CMs) through the dynamic clamp (DC) technique. They found that the optimum compensation of the AP waveform was satisfactory when the Ishihara *I*_K1_ model was used. The subendocardial type of *I*_Kto_ was used (*I*_to1_ in Näbauer et al., 1996 [17]). The open probabilities of individual currents are plotted in Figure 2, Figure 3 and Figure 4. The open probability of whole cell *I*_CaL_ (*p*(O)*_I_*_CaL,X_) was a weighted sum of those distributed in three Ca^2+^ spaces (Xs) in *jnc*, *iz*, and *blk* in the hVC model (Equation (4)).

(4) $$p{\left(O\right)}_{I_{\mathrm{CaL}}}=frcCaL_{jnc}\cdot p{\left(O\right)}_{I_{\mathrm{CaL}},jnc}+frcCaL_{iz}\cdot p{\left(O\right)}_{I_{\mathrm{CaL}},iz}+frcCaL_{blk}\cdot p{\left(O\right)}_{I_{\mathrm{CaL}},blk}$$

where *frcCaL*_X_ represents the fraction of *I*_CaL_ channel distribution in X space (X: *jnc*, *iz,* and *blk*). 

For calculating each current component in the hVC, abbreviations, physical constants, ionic composition of external solution, parameters, and initial values of the model components, see model equations in Supporting Materials and Himeno et al. (2015) [15] or Asakura et al. (2014) [14]. The dimensions of variables in the model equation were usually given as time ms, length µm, concentration mM (except [Ca^2+^]_i_ µM for contraction), electric potential mV, conductance nS, and current pA, unless otherwise described. In the figures for membrane ionic currents in the present paper, the standard pA/pF is used.

The ordinary differential equations (ODE) of the hVC model [15] were time-integrated using the Heun method or Euler method. Computer programs were coded in Visual Basic and run on Visual studio. The time step of integration was set at 0.01 ms, approximately half of the maximum interval, which gives results consistent with those obtained using smaller step sizes. In some preliminary simulations, the Adams–Bashforth and Adams–Moulton integration methods were used to validate the numerical integration using simple methods.

### 4.2. One-Dimensional Strand of the hVC Model

**Figure 5:**
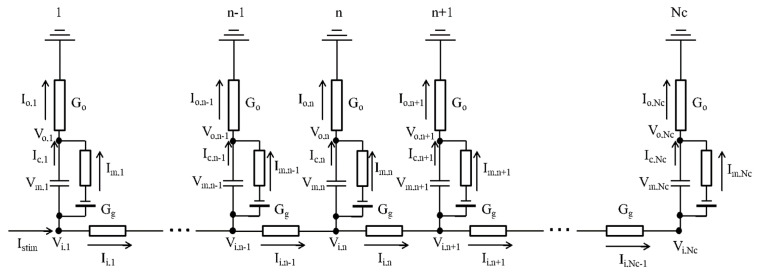
The electrically equivalent circuit of the myocyte strand. *G*_o_; the extracellular conductance of 10 µS, *V*_o_; extracellular potential near the cell surface, *C*_m_; membrane capacitance of 192.5 pF, *V*_m_; membrane potential, *V*_i_; intracellular potential, *I*_o_; extracellular current, *I*_c_; capacitor current, *I*_m_; membrane current, *I*_i_; gap junction current, *G*_g_; the gap junction conductance of 1.5 µS assumed for the end-to-end junction along the longitudinal axis of the cuboid shape myocyte.

Figure 5 shows the electrical coupling between two neighboring electric circuits in the myocyte model in a one-dimensional strand of *N*_c_ (=400) myocytes. The intracellular potential (*V*_i_) was defined as the sum of the extracellular potential (*V*_o_) and the membrane potential (*V*_m_) of each myocyte (Equation (5)).

(5) $$V_{i,n}=V_{o,n}+V_{m,n}$$

The velocity of propagation along the fiber orientation was determined from the slope of the relationship between time and location of the point where the sign of the *V*_o_ was inverted. 

### 4.3. Calculating V_o_ Outside the Myocyte Membrane in the Strand

The equivalent electric circuit of the myocyte strand is shown in Figure 5. The electrically equivalent circuit of the myocyte. Note that this myocyte strand model is different from the bidomain model because all myocytes within the strand have direct contact with the ground through an extracellular medium that has high conductance. This circuit represented experimental conditions of small multicellular preparations soaked in the bath solution, as in the classical experimental studies [5], rather than myocyte sheets tightly packed in the intact heart. In the presented model of the myocyte strand, the *V*_o_ of individual myocyte models was determined from individual *V*_m_, *G*_o,_ and *G*_g_ according to the algorithm as described in Algorithm 1.
**Algorithm 1.** Summary of the calculation for the 1D myocyte strand model.**Step1**: Calculate conductance matrix of A, B, C in Equation (9);**Step2**: Calculate the inverse matrix of the conductance matrix of A, B, C;**Step3**: Calculate the right-hand side of Equation (9) from $V_{m,n}^{t}$;**Step4**$:\;\mathrm{Calculate}\;V_{o,n}^{t}$ in Equation (9) by using the inverse matrix obtained in Step 2;**Step5**$:\;\mathrm{Calculate}\;V_{i,n}^{t}$$\;\mathrm{from}\;V_{o,n}^{t}$$\;\mathrm{and}\;V_{m,n}^{t}$ using Equation (5);**Step6**$:\;\mathrm{Calculate}\;I_{i,n}^{t}$$\;\mathrm{from}\;V_{i,n}^{t}$$\;\mathrm{and}\;V_{i,n+1}^{t}$ using Equation (6);**Step7**$:\;\mathrm{Calculate}\;I_{o,n}^{t}$$\;\mathrm{from}\;I_{i,n}^{t}$$\;\mathrm{and}\;I_{i,n-1}^{t}$ using Equation (7);**Step8**$:\;\mathrm{Determine}\;\frac{dV_{m,n}^{t}}{dt}$$\;\mathrm{using}\;\mathrm{total}\;\mathrm{membrane}\;\mathrm{current}\;I_{m,n}^{t}$$\;\mathrm{and}\;I_{o,n}^{t}$                         $\frac{dV_{m,n}^{t}}{dt}=\frac{I_{c,n}^{t}}{C_{m}}=\frac{-\left(I_{m,n}^{t}-I_{o,n}^{t}\right)}{C_{m}}$;**Step9**: Integration of parameters for the next time step                         $V_{m,n}^{t+dt}=V_{m,n}^{t}+\frac{dV_{m,n}^{t}}{dt}\cdot dt$;**Step10**: Go to Step3.

The current along the longitudinal axis (across the gap junction, *I*_i,n_) was determined by Equation (6), which deals with the *G*_g_ and the voltage difference across the *G*_g_.

(6) $$I_{i,n}=G_{g}\cdot \left(V_{i,n}-V_{i,n+1}\right)=G_{g}\cdot \left(\left(V_{o,n}+V_{m,n}\right)-\left(V_{o,n+1}+V_{m,n+1}\right)\right)$$

The intercellular current along the transverse axis (*I*_o_) is driven by the difference between *I*_i,n−1_ and *I*_i,n_ through *G*_o_.

(7) $$I_{o,n}=I_{i,n-1}-I_{i,n}=G_{o}\cdot V_{o,n}$$

Replacing *I* in Equation (7) with the potentials (*V*_o_, *V*_m_) provides an equation composed of *V* and *G*,

(8) $$-G_{g}\cdot V_{o,n-1}+\left(G_{o}+2\cdot G_{g}\right)\cdot V_{o,n}-G_{g}\cdot V_{o,n+1}=G_{g}\cdot \left(\left(V_{m,n-1}-V_{m,n}\right)-\left(V_{m,n}-V_{m,n+1}\right)\right)$$

which is then rearranged for *V*_o_ of three sequential myocyte models (*V*_o,n−1_, *V*_o,n_, and *V*_o,n+1_) as a function of voltages (*V*_o_ and *V*_m_) as follows;

(9) $$A\cdot V_{o,n-1}+B\cdot V_{o,n}+C\cdot V_{o,n+1}=D\cdot V_{m,n-1}+E\cdot V_{m,n}+F\cdot V_{m,n+1}$$

here, the *V*_m_ of each unit is obtained beforehand by integrating the whole family of membrane currents to calculate the charge across the membrane capacitance. In brief, values of *V*_o_ for all myocytes are calculated by the parallel linear relations of all myocytes using a homemade solver every time after the numerical integration of the variables of individual myocytes. *V*_o_ was assigned a positive sign when *I*_o_ was outward whereas *V*_o_ was assigned a negative sign when *I*_o_ was inward and vice versa. As the *G*_o_ (= 10 µS) was larger than the *G*_g_ (= 1.5 µS), the conduction velocity was largely dependent on *G*_g_. The *G*_o_ should be a scalable parameter, adjusted to the real size and dimension of the external electrode used for measurements. For a tentative *G*_o_ magnitude, the recordings of the QRS complex using the epicardial electrogram [27] were referred to. With a *G*_o_ of 5 µS, full-sized experimental *V*_o_ deflections of 5~10 mV were obtained with a standard maximum rate of rise of 250 V/s. 

The gap junction current (*I*_gap_) was calculated on the right side of the *n*th equivalent circuit.

(10) $$I_{\mathrm{gap}}=I_{i,n}=G_{g}\cdot \left[\left(V_{i,n}-V_{i,n+1}\right)+E_{K,n}\right]$$

To calculate changes in [K^+^]_i_, *I*_gap_ was assumed to be carried purely by K^+^. Thus, the Nernst equilibrium potential *E*_K_ was determined across the gap junction.

(11) $$E_{K,n}=\frac{RT}{F}\cdot \ln\left(\frac{{\left[K^{+}\right]}_{n}}{{\left[K^{+}\right]}_{n+1}}\right)$$

### 4.4. Electrical Stimulation to the Myocyte Model and the Myocyte Strand Model

The conditioning AP was evoked by applying a 5 ms current pulse of −12 pA/pF (K^+^-mediated current) in the single myocyte experiment. In the 1D strand model experiment, the stimulus current was magnified to −35 pA/pF in amplitude and 30 ms in duration to evoke the conditioning AP, which propagated throughout the axis to the right end of the strand. 

To confirm whether the threshold phenomenon is observed in the single hVC model, hyperpolarizing voltage clamp pulses of 5 ms in duration at various test potential (*V*_t_) levels in hyperpolarization were applied during the AP plateau phase. The compensation current given by Equation (12) was applied at various selected time points during the AP plateau phase.

(12) $$I_{\mathrm{compensation}}=-\left(V_{t}-V_{m}\right)\cdot \frac{1}{fb_{\mathrm{gain}}}\;$$

where *fb*_gain_ was 1/(10 × dt), dt = 0.01 ms. 

To trigger the propagation of repolarization in the 1D strand of myocytes, a trigger current pulse of 400 pA/pF and 40 ms in duration was applied simultaneously to 100 myocytes (cell No. 50~150). 

These stimulus currents as well as *I*_compensation_ were added to the total membrane current *I*_m,n_ of the myocyte model, which is used to calculate d*V*_m_/dt in each myocyte. The propagation of repolarization started from the border of the segment of the 100 myocytes. The movement of the wave of propagation was monitored by plotting the *V*_o_ in the 2D display (the myocyte number abscissa and the time ordinate).

### 4.5. Induction of the Second Stable Equilibrium V_m_ in Myocyte Model within the In Silico 1D Strand

The propagation of repolarization could be observed in a straightforward manner if the myocyte strand was depolarized to a stable equilibrium potential near the plateau potential around −5 mV. This was achieved by removing the slow *V*_m_-dependent inactivation of *I*_NaL_ from the state transition scheme, described later in Figure 3D. The equilibrium potential was examined by recording the steady-state current–voltage relationship (*I*-*V* relationship) by applying long voltage clamp pulses of 4.9 s in duration. After confirming that the time-dependent changes were virtually saturated near the end of clamp pulses, the current amplitude was measured. Influences of varying the *I*_NaL_ were examined by changing the fractional amplitude of the fixed I_s_ state of *I*_NaL_.

## Figures and Tables

**Table 1:** The rate of propagation of repolarization in the strand model. The sign ‘F’ indicates that the stimulus failed to evoke the propagation of repolarization. Note that increasing the fraction of I_2_ in (I_2_ + I_s_) by 10 or 20% decreases *G*_NaL_.

	0.639 (−20%)	0.594 (−10%)	Relative *G*_NaL_ 0.549 after Fixing (I_2_ + I_s_)	0.504 (+10%)	0.459 (+20%)
−10%	F	F	F	F	F
control *G*_K1_	F	F	F	1.69	2.11
+10%	F	1.52	1.94	2.38	2.94
+20%	1.79	2.114	2.40	2.69	3.04

**Table 2:** The rate of propagation of excitation and repolarization calculated by varying gap junction conductances (*G*_g_) in the strand model.

*G*_g_ (nS)	1000	1500	2000	2500
rate excitation propagation (cm/s)	36.5	48.0	51.79	58.5
rate repolarization propagation (cm/s)	1.93	2.43	2.60	2.94

## Data Availability

The data that supports the findings of this study are available in the Supplementary Material (Tables S1–S8 and Schemes S1–S6) of this article.

## Supplementary Materials

The following supporting information can be downloaded at: https://www.mdpi.com/article/10.3390/ijms242015378/s1. Supplementary materials include model equations and computer program codes. References [14,15,24,28,29,30,31,32,33,34,35,36,37] are cited in the supplementary materials.
