# Supplementary material for: Ionic Mechanisms of Propagated Repolarization in a One-Dimensional Strand of Human Ventricular Myocyte Model
Source: Int J Mol Sci. 2023 Oct 19;24(20):15378. doi: 10.3390/ijms242015378 (PMC10607672; doi:10.3390/ijms242015378)
Supplement: Supplementary file 1 [file ijms-24-15378-s001.zip › SupplementalMaterials_Himeno_et_al.pdf]

Supplemental Materials

Ionic Mechanisms of Propagated Repolarization in a One-Dimensional Strand of Human Ventricular Myocyte Model

Yukiko Himeno [1]*, Yixin Zhang [1], Suzuka Enomoto [1], Hiroto Nomura [1], Natsuki Yamamoto [1], Shotaro Kiyokawa [1], Mirei Ujihara [1], Yuttamol Muangkram [1], Akinori Noma [1] and Akira Amano [1]

[1] Department of Bioinformatics, College of Life Sciences, Ritsumeikan University, Japan

<contents>

1. Abbreviations

2. Model parameters
   Physical contents
   Ion concentrations
   Substrates
   Cell volume
   Ca2+ buffer
   Boundary Ca2+ diffusion

3. Rate of change in the membrane potential and ion concentrations
   Membrane potential
   Ion concentrations

4. Ion channels and transporters
   GHK equation
   L-type Ca^{2+} current (I_{CaL},LCC)
   Sodium current (I_{Na})
   Inward rectifier potassium current (I_{K1})
   Delayed rectifier K^+ current, fast component (I_{Kr})
   Delayed rectifier K^+ current, slow component (I_{Ks})

Transient outward K^+ current (I_{Kto})
Time-independent currents
Na^+/K^+ pump current (I_{NaK})
Na^+/Ca^+ exchange current (I_{NCX})
Plasma membrane Ca2+-ATPase current (IPMCA)
CaRU
Sarcoplasmic reticulum Ca^{2+} pump (SERCA) current (JSERCA)

5. Contraction
Dynamics of the eCB elongation(h)
Developed tension
Calculation of state transitions of the model

6. Metabolic Parameters

7. Initial values of HuVEC model

8. References

1. Abbreviations

Table S1. Abbreviations in model equations

Membrane excitation	
V_m	membrane potential (mV)
I_{tot_cell}	total current of ion channels and exchangers (pA/pF)
$I_{tot\_X\_a}$	total current of ion 'X' channels and exchangers at space 'a' (pA/pF)
I_{app_blk}	current applied through a patch electrode (pA/pF)
E_X	reversal potential of ion 'X' , determined from the slope conductance of I_X-V_m relationship, or by the Nernst equation (mV)
C_m	membrane capacitance (pF)
G_I	conductance of current 'I' (pA/mV/pF)
GHK_{X_a}	a modified Goldman-Hodgkin-Katz equation of ion 'X' at a space 'a' (mM)
k, α, β, ν	rate constant (/ms)
K_{d_X}	dissociation constant for ion 'X' (/mM)
$P_{I(\_X)}$	converting factor of current 'I' from GHK_X (pA/mM/pF)
v_{cyc_T}	turnover rate of transporter 'T' (/ms)
$p(S)_{(\_a)}$	probability of state 'S' in a scheme of state transitions at a space 'a'
$p(O)_{I(\_a)}$	open probability of current 'I' at space 'a'
V_X	total volume of space 'X' (μm^3)
$[X_{total}]_a$	total concentration of substance 'X' at space 'a' (mM)
$[X_{free}]_a$	free concentration of substance 'X' at space 'a' (mM)
$[X]_a$	concentration of ion 'X' at space 'a' (mM)
J_X	total flux of ion 'X' (amol/ms)
z_X	valence of ion 'X'
$\frac{d[X]_a}{dt}$	rate of change of 'X' concentration at space 'a' (mM/ms)

2. Model parameters

Physical constants

Table S2. Physical constants

R	8.3143	C ・mV/mmol/K
T	310	K
F	96.4867	C/mmol

Ion concentrations

Table S3. Ionic composition of external solution

$[K^+]_o$	4.5	mM
$[Na^+]_o$	140	mM
$[Ca^{2+}]_o$	1.8	mM

Substrates

TableS4. Substrates

$[H^+]_{cyt}$	0.0001	mM
$[Mg^{2+}]_{cyt}$	0.8	mM
$[SPM]$	5	mM

Cell volume

Table S5. Cell compartments

Cell configuration (V_{cell})	$120 \cdot 37.62 \cdot 8.4$	fL
Bulk space (V_{blk})	$0.68 \cdot V_{cell}$	fL
Intermediate zone (V_{iz})	$0.035 \cdot V_{cell}$	fL
Junction space (V_{jnc})	$0.008 \cdot V_{cell}$	fL
Cytsol volume (V_{cyt})	$0.723 \cdot V_{cell}$	fL
Total SR space (V_{SRt})	$0.06 \cdot V_{cell}$	fL
SR releasing site volume (V_{SRrl})	$0.2 \cdot V_{SRt}$	fL
SR uptake site volume (V_{SRup})	$0.8 \cdot V_{SRt}$	fL
Input capacitance (C_m)	192.46	pF

Ca^{2+} buffer

The detailed set of buffer species (1) used in the GPB model was adopted after several simplifications as described in our previous paper (2). In short, we deleted the myosin, Na^+ and Mg^{2+} buffers, and fixed [Mg^{2+}]. The low affinity binding of Ca^{2+} to troponin (TnCl) was replaced by a contraction model (3) and the amount of the high affinity site (TnCh) was adjusted.

Table S6. Ca^{2+} buffer

CaM	calmodulin (mM)
$CaMCa$	CaM-calcium complex (mM)
$[B_{total}CaM]$	total buffer concentration of CaM, 0.0216 (mM)

$TnCh$	troponin C with a high affinity
$TnChCa$	TnCh-calcium complex
$[B_{total}TnCh]$	total buffer concentration of TnCh, 0.108 (mM)
$SRCa$	calcium buffer by SR membrane-Calcium complex
$[B_{total}SR]$	total buffer concentration on SR membrane, 0.01539 (mM)
$[Ca^{2+}_{total}]_{SRrl}$	total calcium concentration in release site of the SR (mM)
$[Ca^{2+}]_{SRrl}$	free calcium concentration in release site of the SR (mM)
$[B_{total}H]_{iz}$	total sarcolemmal calcium buffer concentration with a high affinity in iz space, 0.2178 (mM)
$[B_{total}L]_{iz}$	total sarcolemmal calcium buffer concentration with a low affinity in iz space, 0.6078 (mM)
$[B_{total}H]_{jnc}$	total sarcolemmal calcium buffer concentration with a high affinity in jnc space, 0.398 (mM)
$[B_{total}L]_{jnc}$	total sarcolemmal calcium buffer concentration with a low affinity in jnc space, 1.1095 (mM)
L_b	calcium-sarcolemmal buffer complex with a low affinity
H_b	calcium-sarcolemmal buffer complex with a high affinity
$B_{total}CSQN$	total buffer concentration of calsequestrin, 3.0 (mM)

Bulk space (*blk*)

$$\frac{d[CaMCa]}{dt} = k_{on\_CaM} \cdot [Ca^{2+}]_{blk} \cdot ([B_{total}CaM] - [CaMCa]) - k_{off\_CaM} \cdot [CaMCa]$$

$k_{off\_CaM} = 0.238, k_{on\_CaM} = 34$

$$\frac{d[TnChCa]}{dt} = k_{on\_TnCh} \cdot [Ca^{2+}]_{blk} \cdot ([B_{total}TnCh] - [TnChCa]) - k_{off\_TnCh} \cdot [TnChCa]$$

$k_{off\_TnCh} = 0.000032, k_{on\_TnCh} = 2.37$

$$\frac{d[SRCa]}{dt} = k_{on\_SR} \cdot [Ca^{2+}]_{blk} \cdot ([B_{total}SR] - [SRCa]) - k_{off\_SR} \cdot [SRCa]$$

$k_{off\_SR} = 0.06, k_{on\_SR} = 100$

Intermediate zone (*iz*)

$$[L_{free}]_{iz} = \frac{[B_{total}L]_{iz}}{1+\frac{[Ca^{2+}]_{iz}}{K_{dL\_iz}}}$$

$K_{dL\_iz} = \frac{k_{off\_L\_iz}}{k_{on\_L\_iz}}, k_{off\_L\_iz} = 1.3, k_{on\_L\_iz} = 100$

$$[H_{free}]_{iz} = \frac{[B_{total}H]_{iz}}{1+\frac{[Ca^{2+}]_{iz}}{K_{dH\_iz}}}$$

$K_{dH\_iz} = \frac{k_{off\_H\_iz}}{k_{on\_H\_iz}}, k_{off\_H\_iz} = 0.03, k_{on\_H\_iz} = 100$

$$[Ca^{2+}]_{iz} = \frac{[Ca_{tot}]_{iz}}{1+\frac{[L_{free}]_{iz}}{K_{dL\_iz}}+\frac{[H_{free}]_{iz}}{K_{dH\_iz}}}$$

Junctional space (*jnc*)

$$[L_{free}]_{jnc} = \frac{[B_{total}L]_{jnc}}{1+\frac{[Ca^{2+}]_{jnc}}{K_{dL\_jnc}}}$$

$$K_{dL\_jnc} = \frac{k_{off\_L\_jnc}}{k_{on\_L\_jnc}}, k_{off\_L\_jnc} = 1.3, k_{on\_L\_jnc} = 100$$

$$[H_{free}]_{jnc} = \frac{[B_{total}H]_{jnc}}{1+\frac{[Ca^{2+}]_{jnc}}{K_{dH\_jnc}}}$$

$$K_{dH\_jnc} = \frac{k_{off\_H\_jnc}}{k_{on\_H\_jnc}}, k_{off\_H\_jnc} = 0.03, k_{on\_H\_jnc} = 100$$

$$[Ca^{2+}]_{jnc} = \frac{[Ca_{tot}]_{jnc}}{1+\frac{[L_{free}]_{jnc}}{K_{dL\_jnc}}+\frac{[H_{free}]_{jnc}}{K_{dH\_jnc}}}$$

Release site of the SR(*SRrl*)

$$K_{d\_CSQN\_Ca} = \frac{k_{off\_CSQN}}{k_{on\_CSQN}}$$

$$k_{off\_CSQN} = 65, k_{on\_CSQN} = 100$$

$$a = 1$$

$$b = [B_{total}CSQN] - [Ca^{2+}_{total}]_{SRrl} + K_{d\_CSQN\_Ca}$$

$$c = -K_{d\_CSQN\_Ca} \cdot [Ca^{2+}_{total}]_{SRrl}$$

$$[Ca^{2+}]_{SRrl} = \frac{-b+\sqrt{b^2-4\cdot a\cdot c}}{2\cdot a}$$

Boundary Ca^{2+} diffusion

Ca^{2+} transfer between cytosolic compartments

$$J_{Ca\_jnciz} = G_{dCa\_jnciz} \cdot ([Ca^{2+}]_{jnc} - [Ca^{2+}]_{iz})$$

$$G_{dCa\_jnciz} = 3395.88 \cdot fL \cdot ms^{-1}$$

$$J_{Ca\_izblk} = G_{dCa\_izblk} \cdot ([Ca^{2+}]_{iz} - [Ca^{2+}]_{blk})$$

$$G_{dCa\_jzblk} = 3507.78 \cdot fL \cdot ms^{-1}$$

Ca^{2+} transfer from SR uptake site to release site

$$J_{trans\_SR} = P_{trans} \cdot ([Ca^{2+}]_{SRup} - [Ca^{2+}]_{SRrl})$$

$$P_{trans} = 4.8037$$

3. Rate of change in the membrane potential and ion concentrations

Membrane potential

$$\frac{dV_m}{dt} = -(I_{tot\_cell} + I_{app}) \quad \text{(Eq. S1)}$$

$$I_{tot\_cell} = I_{tot\_Na} + I_{tot\_Ca} + I_{tot\_K} \quad \text{(Eq. S2)}$$

$$I_{tot\_Ca} = I_{tot\_Ca\_jnc} + I_{tot\_Ca\_iz} + I_{tot\_Ca\_blk} \quad \text{(Eq. S3)}$$

$$I_{tot\_Ca\_jnc} = I_{CaL\_Ca\_LR} + I_{CaL\_Ca\_L0} \quad \text{(Eq. S4)}$$

$$I_{tot\_Ca\_iz} = I_{CaL\_Ca\_iz} + I_{PMCA\_iz} + I_{NCX\_Ca\_iz} + I_{Cab\_iz} \quad \text{(Eq. S5)}$$

$$I_{tot\_Ca\_blk} = I_{CaL\_Ca\_blk} + I_{PMCA\_blk} + I_{NCX\_Ca\_blk} + I_{Cab\_Ca\_blk} \quad \text{(Eq. S6)}$$

$$I_{tot\_Na} = \left(I_{CaL\_Na\_jnc} + I_{CaL\_Na\_iz} + I_{CaL\_Na\_blk}\right) + (\, I_{NCX\_Na\_iz} + I_{NCX\_Na\_blk}) \quad \text{(Eq. S7)}$$
$$+\left(I_{Ks\_Na\_iz} + I_{Ks\_Na\_blk}\right) + I_{NaT\_Na} + I_{NaL\_Na} + I_{NaK\_Na} + I_{Kto\_Na} + I_{bNSC\_Na}$$
$$+(I_{LCCa\_Na\_iz} + I_{LCCa\_Na\_blk})$$

$$I_{tot\_K} = \left(I_{CaL\_K\_jnc} + I_{CaL\_K\_iz} + I_{CaL\_K\_blk}\right) + I_{NaT\_K} + I_{NaL\_K} + I_{K1\_K} + I_{Kr\_K} \quad \text{(Eq. S8)}$$
$$+(I_{Ks\_K\_iz} + I_{Ks\_K\_blk}) + I_{Kto\_K} + I_{Kpl} + I_{NaK\_K} + I_{KATP\_K\_cyt} + I_{bNSC\_K} \quad 9$$
$$+(I_{LCCa\_K\_iz} + I_{LCCa\_K\_blk})$$

Ion concentrations

$$\frac{d[Ca^{2+}_{total}]jnc}{dt} = -\frac{I_{tot\_Ca\_jnc} \cdot C_m}{V_{jnc} \cdot 2 \cdot F} + \frac{J_{Ca\_rel}}{V_{jnc}} - \frac{J_{Ca\_jnciz}}{V_{jnc}} \quad \text{(Eq. S9)}$$

$$\frac{d[Ca^{2+}_{total}]iz}{dt} = -\frac{I_{tot\_Ca\_iz} \cdot C_m}{V_{iz} \cdot 2 \cdot F} + \frac{J_{Ca\_jnciz}}{V_{iz}} - \frac{J_{Ca\_izblk}}{V_{iz}} \quad \text{(Eq. S10)}$$

$$\frac{d[Ca^{2+}_{total}]blk}{dt} = -\frac{I_{tot\_Ca\_blk} \cdot C_m}{V_{blk} \cdot 2 \cdot F} + \frac{J_{Ca\_SERCA}}{V_{blk}} - \frac{J_{Ca\_izblk}}{V_{blk}} \quad \text{(Eq. S11)}$$

$$\frac{d[Ca^{2+}]_{SRup}}{dt} = -\frac{J_{SERCA}}{V_{SRup}} - \frac{J_{trans\_SR}}{V_{SRup}} \quad \text{(Eq. S12)}$$

$$\frac{d[Ca^{2+}_{total}]_{SRrl}}{dt} = -\frac{J_{trans\_SR}}{V_{SRrl}} - \frac{J_{rel\_SR}}{V_{SRrl}} \quad \text{(Eq. S13)}$$

$$\frac{d[Na^{+}]_i}{dt} = -\frac{I_{tot\_Na} \cdot C_m}{V_{cyt} \cdot F} \quad \text{(Eq. S14)}$$

$$\frac{d[K^{+}]_i}{dt} = -\frac{(I_{tot\_K} + (I_{app}) \cdot C_m}{V_{cyt} \cdot F} \quad \text{(Eq. S15)}$$

Free Ca^{2+} concentrations as calculated below are used as intracellular Ca^{2+} concentrations in each compartment.

$$[Ca^{2+}_{free}]_{jnc} = [Ca^{2+}_{total}]_{jnc} - \left([L_{bind}]_{jnc} + [H_{bind}]_{jnc}\right)$$

$$[L_{bind}]_{jnc} = [B_{total}L]_{jnc} - [L_{free}]_{jnc}$$
$$[H_{bind}]_{jnc} = [B_{total}H]_{jnc} - [H_{free}]_{jnc}$$
$$[Ca^{2+}_{free}]_{iz} = [Ca^{2+}_{total}]_{iz} - ([L_{bind}]_{iz} + [H_{bind}]_{iz})$$
$$[L_{bind}]_{iz} = [B_{total}L]_{iz} - [L_{free}]_{iz}$$
$$[H_{bind}]_{iz} = [B_{total}H]_{iz} - [H_{free}]_{iz}$$
$$[Ca^{2+}_{free}]_{blk} = [Ca^{2+}_{total}]_{blk} - \left([CaMCa] + [TnChCa] + [SRCa] + nCaBS \cdot \frac{(cMDP + cAMDPw + cAMDs + cAMT + cMDPt)}{1000}\right)$$

4. Ion channels and transporters

GHK equation

The magnitudes of ion channel currents were described by the ohmic equation or by the GHK equation. In the latter case, the term to convert mM to pA (permeability times zF) in the original GHK equation was represented by a lumped converting factor, P in a unit of pA·mM^{-1}, because of unknown total number of channels within a cell and single channel ion permeability. Then, the fully-activated current amplitude (I) for an ion X was given by,

$$I = P \cdot GHK_X$$

where GHK_X is,

$$GHK_X = \frac{z_X F V_m}{RT} \cdot \frac{\left([X]_i - [X]_o \cdot exp\left(\frac{-z_X F V_m}{RT}\right)\right)}{\left(1 - exp\left(\frac{-z_X F V_m}{RT}\right)\right)}$$

Nernst equation

$$E_X = \frac{R \cdot T}{z_X \cdot F} \cdot log\left(\frac{[X]_o}{[X]_i}\right)$$

L-type Ca^{2+} current (I_{CaL}, LCC)

The model developed by Himeno *et al.*(4) was used after adjusting P_{CaL_Ca}, which was multiplied by 2.1.I_{CaL} is composed of Ca^{2+}, Na^{+} and K^{+} components, which are described with GHK_X for each cation.

$$I_{CaL} = (I_{CaL\_Ca\_jnc} + I_{CaL\_Na\_jnc} + I_{CaL\_K\_jnc}) + (I_{CaL\_Ca\_iz} + I_{CaL\_Na\_iz} + I_{CaL\_K\_iz}) + (I_{CaL\_Ca\_blk} + I_{CaL\_Na\_blk} + I_{CaL\_K\_blk}) \quad \text{(Eq. S16)}$$

$$I_{CaL\_X\_a} = f_{CaL\_a} \cdot P_{CaL\_X} \cdot GHK_{X\_a} \cdot pO_{LCC\_a} \cdot \frac{1}{1+\left(\frac{1.4}{[MgATP\_cyt]}\right)^3} \quad \text{(Eq. S17)}$$

$$pO_{LCC\_a} = Y_{ooo} + Y_{ooc} \qquad a = (blk, iz, jnc)\ X = (Ca, Na, K) \quad \text{(Eq. S18)}$$

fraction of I_{CaL}

$$f_{CaL\_jnc} = 0.75,\ f_{CaL\_blk} = 0.1,\ f_{CaL\_iz} = 0.15$$

Converting factors

$$P_{CaL\_Ca} = 14.21 \cdot C_m$$

$$P_{CaL\_Na} = 0.0000185 \cdot P_{CaL\_Ca}$$

$$P_{CaL\_K} = 0.000367 \cdot P_{CaL\_Ca}$$

Rate constants for V_m-gate(α_+, α_-) and Ca^{2+}-gate($\varepsilon_+, \varepsilon_-$)

$$\alpha_+ = \frac{1}{3.734 \cdot exp\left(-\frac{V_m}{8.5}\right) + 0.35 \cdot exp\left(-\frac{V_m}{3500}\right)} \quad \text{(Eq. S19)}$$

$$\alpha_- = \frac{1}{4.65 \cdot exp\left(\frac{V_m}{15}\right) + 1.363 \cdot exp\left(\frac{V_m}{100}\right)} \quad \text{(Eq. S20)}$$

$$\varepsilon_+ = \frac{[Ca^{2+}]_{nd} \cdot \alpha_+}{T_L \cdot K_L} \quad \text{(Eq. S21)}$$

$$\varepsilon_- = \frac{1}{8084 \cdot exp\left(\frac{Vm}{10}\right) + 158 \cdot exp\left(\frac{Vm}{1000}\right)} + \frac{1}{134736 \cdot exp\left(-\frac{Vm}{5}\right) + 337 \cdot exp\left(-\frac{Vm}{2000}\right)} \quad \text{(Eq. S22)}$$

Sodium current(I_{Na})

The model developed by Asakura *et al.* (2) was used. To facilitate calculation of rapid gating kinetics of the current, integration of the parameters was conducted using Rush-Larsen method(5). The whole cell I_{Na} was given by a sum of I_{NaT} and I_{NaL} with a fraction(f_L) of 0.13125 for I_{NaL}. The current was carried by both Na^+ and K^+ at ratio of permeability, P_{Na}/P_K, of 10.

$$I_{Na} = I_{NaT} + I_{NaL} \quad \text{(Eq. S23)}$$

$$f_L = 0.13125,\ P_{Na} = 8.1072 \cdot C_m\ (pA/mM)$$

Transient component(I_{NaT})

$$I_{NaT} = (1 - f_L) \cdot P_{Na} \cdot (GHK_{Na} + 0.1 \cdot GHK_K) \cdot p(O)_{NaT} \quad \text{(Eq. S24)}$$

$$\frac{dp(C)_{NaT}}{dt} = k_{OC} \cdot p(O)_{NaT} + k_{I2C} \cdot p(I_2)_{NaT} + k_{Isb} \cdot p(I_s)_{NaT} - (k_{Isf} + f_{C\_Na} \cdot (k_{C2O} + k_{C2I2})) \cdot p(C)_{NaT} \quad \text{(Eq. S25)}$$

$$\frac{dp(O)_{NaT}}{dt} = k_{I2O} \cdot p(I_2)_{NaT} + f_{C\_Na} \cdot k_{C2O} \cdot p(C)_{NaT} - (k_{OC} + k_{OI2}) \cdot p(O)_{NaT} \quad \text{(Eq. S26)}$$

$$\frac{dp(I_2)_{NaT}}{dt} = f_{C_{Na}} \cdot k_{C2I2} \cdot p(C)_{NaT} + k_{OI2} \cdot p(O)_{NaT} + k_{Isb} \cdot p(I_s)_{NaT} - \left(k_{I2C} + k_{I2O} + k_{Isf}\right) \cdot p(I_2)_{NaT} \quad \text{(Eq. S27)}$$

$$\frac{dp(I_s)_{NaT}}{dt} = k_{Isf} \cdot p(I_2)_{NaT} + k_{Isf} \cdot p(C)_{NaT} - 2 \cdot k_{Isb} \cdot p(I_s)_{NaT} \quad \text{(Eq. S28)}$$

$$f_{C\_Na} = \frac{C_2}{(C_1 + C_2)} = \frac{1}{1 + exp\left(-\frac{Vm + 48}{7}\right)} \quad \text{(Eq. S29)}$$

$$k_{C2O} = \frac{0.5}{0.0025 \cdot exp\left(\frac{Vm}{-8.0}\right) + 0.15 \cdot exp\left(\frac{Vm}{-100.0}\right)} \quad \text{(Eq. S30)}$$

$$k_{OC} = \frac{0.5}{30.0 \cdot exp\left(\frac{Vm}{12.0}\right) + 0.53 \cdot exp\left(\frac{Vm}{50.0}\right)} \quad \text{(Eq. S31)}$$

$$k_{OI2} = \frac{1}{0.0433 \cdot exp\left(\frac{Vm}{-27.0}\right) + 0.34 \cdot exp\left(\frac{Vm}{-2000.0}\right)} \quad \text{(Eq. S32)}$$

$$k_{I2O} = 0.0001312$$

$$k_{C2I2} = \frac{0.5}{1.0 + \frac{k_{I2O} \cdot k_{OC}}{k_{OI2} \cdot k_{C2O}}} \quad \text{(Eq. S33)}$$

$$k_{I2C} = 0.5 - k_{C2I2} \quad \text{(Eq. S34)}$$

$$k_{Isb} = \frac{1}{300000.0 \cdot exp\left(\frac{Vm}{10.0}\right) + 50000.0 \cdot exp\left(\frac{Vm}{16.0}\right)} \quad \text{(Eq. S35)}$$

$$k_{Isf} = \frac{1}{0.016 \cdot exp\left(\frac{Vm}{-9.9}\right) + 8.0 \cdot exp\left(\frac{Vm}{-45.0}\right)} \quad \text{(Eq. S36)}$$

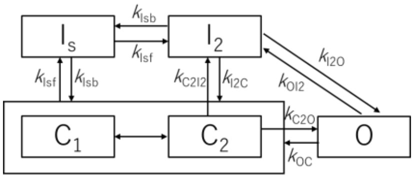

Scheme 1. I_{NaT} model

Late component(I_{NaL})

The k_{I1I2}, k_{OI1}, k_{I1O}, k_{I1C} and k_{C2I1} are specific for I_{NaL}, and other rate constants are the same as in I_{NaL}.

$$I_{NaL} = f_L \cdot P_{Na} \cdot (GHK_{Na} + 0.1 \cdot GHK_K) \cdot p(O)_{NaL} \quad \text{(Eq. S37)}$$

$$\frac{dp(C)_{NaL}}{dt} = k_{OC} \cdot p(O)_{NaL} + k_{I1C} \cdot p(I_1)_{NaL} + k_{I2C} \cdot p(I_2)_{NaL} + k_{Isb} \cdot p(I_s)_{NaL} \quad \text{(Eq. S38)}$$

$$-\left(k_{Isf} + f_{C\_Na} \cdot (k_{C2O} + k_{C2I2} + k_{C2I1})\right) \cdot p(C)_{NaL}$$

$$\frac{dp(O)_{NaL}}{dt} = k_{I1O} \cdot p(I_1)_{NaL} + f_{C\_Na} \cdot k_{C2O} \cdot p(C)_{NaL} - (k_{OC} + k_{OI1}) \cdot p(O)_{NaL} \quad \text{(Eq. S39)}$$

$$\frac{dp(I_1)_{NaL}}{dt} = k_{OI1} \cdot p(O)_{NaL} + f_{C\_Na} \cdot k_{C2I1} \cdot p(C)_{NaL} - (k_{I1O} + k_{I1C} + k_{I1I2}) \cdot p(I_1)_{NaL} \quad \text{(Eq. S40)}$$

$$\frac{dp(I_2)_{NaL}}{dt} = f_{C_{Na}} \cdot k_{C2I2} \cdot p(C)_{NaL} + k_{I1I2} \cdot p(I_1)_{NaL} + k_{Isb} \cdot p(I_s)_{NaL} \quad \text{(Eq. S41)}$$

$$-\left(k_{I2C} + k_{Isf}\right) \cdot p(I_2)_{NaL}$$

$$\frac{dp(I_s)_{NaL}}{dt} = k_{Isf} \cdot p(I_2)_{NaL} + k_{Isf} \cdot p(C)_{NaL} - 2 \cdot k_{Isb} \cdot p(I_s)_{NaL} \quad \text{(Eq. S42)}$$

$k_{I1I2} = 0.00534$

$k_{OI1} = k_{OI2}$

$k_{I1O} = 0.01$

$k_{I1C} = k_{I2C}$

$k_{C2I1} = k_{C2I2}$

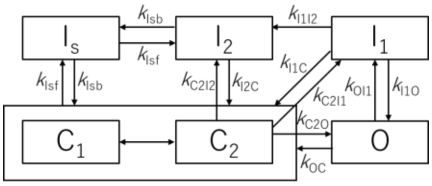

Scheme 2. I_{NaL} model

Inward rectifier potassium current(I_{K1})

The model developed by Yan and Ishihara (6) and Ishihara and Yan (7) was used after adjusting the amplitude of I_{K1} to obtain repolarizing rate of ~1 V/s. The gating in this model was described by two modes: in mode 1, Mg^{2+} and spermine blocks the channel in a competitive manner, while in mode 2, spermine instantaneously blocks the channel in a V_m-dependent

manner.

$$I_{K1} = G_{K1} \cdot x_{K1} \cdot (V_m - E_k) \cdot p(O)_{K1} \qquad G_{K1} = 1.353 \cdot C_m \tag{Eq. S43}$$

$$x_{K1} = \frac{\left(\frac{[K^+]_o}{4.5}\right)^{0.4}}{1.0 + exp\left(-\frac{[K^+]_o - 2.2}{0.6}\right)} \tag{Eq. S44}$$

$$p(O)_{K1} = po_{mode1} + po_{mode2} \tag{Eq. S45}$$

$$po_{mode1} = f_{mode1} \cdot (1 - pb_{spm}) \cdot (po_{Mg} + \frac{2}{3} \cdot po_{Mg1} \cdot \frac{1}{3} \cdot po_{Mg2}) \tag{Eq. S46}$$

$$f_{mode1} = 0.9$$

$$po_{mode2} = \frac{(1 - f_{mode1})}{1.0 + \frac{[SPM]}{40.0 \cdot exp\left(-\frac{V_m - E_k}{9.1}\right)}} \tag{Eq. S47}$$

The Mg2+-block in the mode1

$$po_{Mg} = f_O \cdot f_O \cdot f_O \tag{Eq. S48}$$

$$po_{Mg1} = 3.0 \cdot f_O \cdot f_O \cdot f_B \tag{Eq. S49}$$

$$po_{Mg2} = 3.0 \cdot f_O \cdot f_B \cdot f_B \tag{Eq. S50}$$

$$f_O = \frac{\alpha_{Mg}}{\alpha_{Mg} + \beta_{Mg}}, \; f_B = \frac{\beta_{Mg}}{\alpha_{Mg} + \beta_{Mg}} \tag{Eq. S51}$$

$$\alpha_{Mg} = 12.0 \cdot exp(-0.025 \cdot (V_m - E_k)) \tag{Eq. S52}$$

$$\beta_{Mg} = 28 \cdot [Mg^{2+}]_{cyt} \cdot exp(0.025 \cdot (V_m - E_k)) \tag{Eq. S53}$$

The SPM-block in the mode2

$$\frac{dPb_{spm}}{dt} = \beta_{SPM} \cdot po_{Mg} \cdot (1 - Pb_{spm}) - \alpha_{SPM} \cdot Pb_{spm} \tag{Eq. S54}$$

$$\alpha_{SPM} = \frac{0.17 \cdot exp(-0.07 \cdot (V_m - E_k) + 8[Mg^{2+}]_{cyt}))}{1.0 + 0.01 \cdot exp(0.12 \cdot ((V_m - E_k) + 8[Mg^{2+}]_{cyt}))} \tag{Eq. S53}$$

$$\beta_{Mg} = \frac{0.28 \cdot [SPM] \cdot exp(0.15 \cdot (V_m - E_k) + 8[Mg^{2+}]_{cyt}))}{1.0 + 0.01 \cdot exp(0.13 \cdot ((V_m - E_k) + 8[Mg^{2+}]_{cyt}))} \tag{Eq. S54}$$

Delayed rectifier K+ current, fast component (I_{Kr})

The model was taken from Asakura *et al.* (2) was used after adjusting the conductance of I_{Kr}, G_{Kr}. The current amplitude is described with an ohmic equation.

$$I_{Kr} = G_{Kr} \cdot (V_m - E_K) \cdot p(O)_{Kr} \cdot \left(\frac{[K^+]_o}{4.5}\right)^{0.2} \tag{Eq. S55}$$

$$G_{Kr} = 0.02 \cdot 0.83 \cdot 1.6 \cdot C_m$$

The open probability of the channel is described with three gating parameters, y_1, y_2 and y_3.

$$p(O)_{Kr} = (0.6 \cdot y_1 + 0.4 \cdot y_2) \cdot y_3 \tag{Eq. S56}$$

$$\frac{dy_1}{dt} = \alpha_{y1} \cdot (1.0 - y_1) - \beta_{y1} \cdot y_1 \tag{Eq. S57}$$

$$\frac{dy_2}{dt} = \alpha_{y2} \cdot (1.0 - y_2) - \beta_{y2} \cdot y_2 \tag{Eq. S58}$$

$$\frac{dy_3}{dt} = \alpha_{y3} \cdot (1.0 - y_3) - \beta_{y3} \cdot y_3 \tag{Eq. S59}$$

$$\alpha_{y1} = \frac{1.0}{35.0 \cdot exp\left(-\frac{Vm}{10.5}\right) + 75.0 \cdot exp\left(-\frac{Vm}{100.0}\right)} \quad \text{(Eq. S60)}$$

$$\beta_{y1} = \frac{1.0}{470.0 \cdot exp\left(\frac{Vm}{8.3}\right) + 220.0 \cdot exp\left(\frac{Vm}{29.0}\right)} \quad \text{(Eq. S61)}$$

$$\alpha_{y2} = \frac{1.0}{350.0 \cdot exp\left(-\frac{Vm}{10.5}\right) + 300.0 \cdot exp\left(-\frac{Vm}{100.0}\right)} \quad \text{(Eq. S62)}$$

$$\beta_{y2} = \frac{1.0}{1850.0 \cdot exp\left(\frac{Vm}{8.3}\right) + 2200.0 \cdot exp\left(\frac{Vm}{29.0}\right)} \quad \text{(Eq. S63)}$$

$$\alpha_{y3} = \frac{1.0}{0.015 \cdot exp\left(\frac{Vm}{6.0}\right) + 7.0 \cdot exp\left(\frac{Vm}{60.0}\right)} \quad \text{(Eq. S64)}$$

$$\beta_{y3} = \frac{1.0}{0.114 \cdot exp\left(-\frac{Vm}{9.2}\right) + 2.3 \cdot exp\left(-\frac{Vm}{1000.0}\right)} \quad \text{(Eq. S65)}$$

Delayed rectifier K+ current, slow component (I_{Ks})

The model developed by Asakura *et al.*(2) was used after adjusting P_{Ks_K}. I_{Ks} is composed of K^+ and Na^+ components described with the modified GHK equations. The permeability ratio is $P_{Na}/P_K=0.04$.

$$I_{Ks} = (I_{Ks\_K\_iz} + I_{Ks\_Na\_iz}) + (I_{Ks\_K\_blk} + I_{Ks\_Na\_blk}) \quad \text{(Eq. S66)}$$

$$I_{Ks\_X\_a} = f_{Ks\_a} \cdot P_{Ks\_X} \cdot GHK_X \cdot p(O)_{Ks\_a} \qquad a = (blk,\ iz),\ \ X = (K, Na) \quad \text{(Eq. S67)}$$

Converting factors

$$P_{Ks\_K} = 0.9$$

$$P_{Ks\_Na} = 0.04 \cdot P_{Ks\_K}$$

Ten percent of the channel population is distributed to *jnc*, and the rest in *blk*.

$$f_{Ks\_iz} = 0.1$$

$$f_{Ks\_blk} = 0.9$$

$$p(O)_{Ks\_a} = (p(O_v))^2 \cdot (0.99 \cdot p_a(O_c) + 0.01) \quad \text{(Eq. S68)}$$

A basal active fraction of 1% was assumed in the Ca^{2+} gate.

Voltage gate

$$\frac{dp(O_v)}{dt} = \alpha_1 \cdot (1.0 - p(O_v)) - \beta_1 \cdot p(O_v) \quad \text{(Eq. S69)}$$

$$\alpha_1 = \frac{1.0}{150.0 \cdot exp\left(-\frac{Vm}{25.0}\right) + 900.0 \cdot exp\left(-\frac{Vm}{200.0}\right)} \quad \text{(Eq. S70)}$$

$$\beta_1 = \frac{1.0}{1000.0 \cdot exp\left(\frac{Vm}{13.0}\right) + 220.0 \cdot exp\left(\frac{Vm}{50.0}\right)} \quad \text{(Eq. S71)}$$

Calcium gate

The Ca^{2+}-dependent activation gate was represented with a three-state scheme; sequential C_1, C_2 and O_c states. Ca^{2+}-binding reaction was couples with the transition from C_2 to O_c. α_{2a} was slightly changed from 2.24.

$$\frac{dp_a(O_c)}{dt} = \alpha_{2a} \cdot p_a(C_2) - \beta_2 \cdot p_a(O_c) \quad \text{(Eq. S72)}$$

$$\frac{dp_a(C_2)}{dt} = \alpha_3 \cdot p_a(C_1) - \beta_3 \cdot p_a(C_2) - \alpha_{2a} \cdot p_a(C_2) + \beta_2 \cdot p_a(O_c) \quad \text{(Eq. S73)}$$

$$p_a(C_1) = 1 - p_a(C_2) - p_a(O_c) \quad a = (blk, iz) \quad \text{(Eq. S74)}$$

$\alpha_{2a} = 2.25 \cdot [Ca^{2+}]_a$

$\beta_2 = 0.000296$

$\alpha_3 = 0.0003$

$\beta_3 = 0.03$

Transient outward K^+ current(I_{Kto})

The model was taken from Asakura *et al.* (2) was used after adjusting P_{Kto_K}.

The magnitude of I_{Kto} is composed of K^+ and Na^+ components described with GHK and a pNa/pK ratio = 0.09. The conductance as well as the gating were determined separately for the end(*Endo*)- and epi(*Epi*)-cardium. In the present study, the *Endo* model was used. Replacement by *Epi* I_{Kto} model for the *Endo* type in the cell model well reconstructed the *Epi* AP(not shown).

$$I_{Kto} = I_{Kto\_Na} + I_{Kto\_K} \quad \text{(Eq. S75)}$$

$$I_{Kto\_X} = P_{Kto\_X} \cdot GHK_x \cdot p(O)_{Kto} \quad X = (K, Na) \quad \text{(Eq. S76)}$$

Converting factors in Asakura *et al.* (2014) model were multiplied by 0.73.

$$P_{Kto\_K} = 0.01825 \cdot 0.73(Endo) \cdot C_m, \quad 0.08553 \cdot 0.73 \cdot C_m(Epi)$$

$$P_{Kto\_Na} = 0.09 \cdot P_{Kto_K}(End\ and\ Epi)$$

The gating is determined by an activation gate (*y1*) and inactivation gate (*y2*), both of which are described by the two-state gating scheme.

$$p(O)_{Kto} = y_{1Kto} \cdot y_{2Kto} \quad \text{(Eq. S77)}$$

$$\frac{dy_{1Kto}}{dt} = \alpha_{y1Kto} \cdot (1.0 - y_{1Kto}) - \beta_{y1Kto} \cdot y_{1Kto} \quad \text{(Eq. S78)}$$

$$\frac{dy_{2Kto}}{dt} = \alpha_{y2Kto} \cdot (1.0 - y_{2Kto}) - \beta_{y2Kto} \cdot y_{2Kto} \quad \text{(Eq. S79)}$$

Endocardium

$$\alpha_{y1Kto} = \frac{1.0}{9.0 \cdot exp\left(-\frac{V_m}{20.0}\right)} \quad \text{(Eq. S80)}$$

$$\beta_{y1Kto} = \frac{1.0}{2.1 \cdot exp\left(\frac{V_m}{60.0}\right)} \quad \text{(Eq. S81)}$$

$$\alpha_{y2Kto} = \frac{1.0}{950.0 \cdot exp\left(\frac{V_m}{500.0}\right)} \quad \text{(Eq. S83)}$$

$$\beta_{y2Kto} = \frac{1.0}{40.0 \cdot exp\left(-\frac{V_m}{9.0}\right)} + 13.0 \cdot exp\left(-\frac{V_m}{1000.0}\right) \quad \text{(Eq. S84)}$$

Epicardium

$$\alpha_{y1Kto} = \frac{1.0}{9.0 \cdot Exp\left(-\frac{V_m}{20.0}\right)} \quad \text{(Eq. S86)}$$

$$\beta_{y1Kto} = \frac{1.0}{2.1 \cdot Exp\left(\frac{V_m}{36.5}\right)} \quad \text{(Eq. S87)}$$

$$\alpha_{y2Kto} = \frac{1.0}{365.0 \cdot Exp\left(\frac{V_m}{8.65}\right) + 9.4 \cdot Exp\left(\frac{V_m}{5000.0}\right)} \quad \text{(Eq. S88)}$$

$$\beta_{y2Kto} = \frac{1.0}{4.0 \cdot Exp\left(-\frac{V_m}{8.5}\right) + 8.0 \cdot Exp\left(-\frac{V_m}{5000.0}\right)} \quad \text{(Eq. S89)}$$

Time-independent current

All equations for these currents were taken from Takeuchi *et al.* (8) as described in Asakura *et al.*(2)

Voltage-dependent potassium current (plateau current)(I_{Kpl})

P_{Kpl} in the original model was multipled by 0.4.

$$I_{Kpl} = P_{Kpl} \cdot p(O)_{kpl} \cdot GHK_K \quad \text{(Eq. S90)}$$

$$P_{Kpl} = 0.000043 \cdot 0.4 \cdot C_m \cdot \left(\frac{[K^+]_o}{5.4}\right)^{0.16} \quad \text{(Eq. S91)}$$

$$p(O)_{kpl} = \frac{V_m}{1 - exp\left(-\frac{V_m}{13.0}\right)} \quad \text{(Eq. S92)}$$

Background calcium current(I_{Cab})

P_{Cab} in the original model was multipled by 0.1.

$$I_{Cab} = I_{Cab\_iz} + I_{Cab\_blk} \quad \text{(Eq. S93)}$$

$$I_{Cab\_a} = P_{Cab\_a} \cdot f_{Cab\_a} \cdot GHK_{Ca}, \quad a = (blk,\ iz) \quad \text{(Eq. S94)}$$

$$P_{Cab\_a} = 0.0006822 \cdot 0.1 \cdot C_m$$

Fraction of I_{Cab}

$f_{Cab\_iz} = 0.1, \quad f_{Cab\_blk} = 0.9$

Background non-selective cation current(I_{bNSC})

$$I_{bNSC} = P_{bNSC\_K} + P_{bNSC\_Na} \quad \text{(Eq. S95)}$$

$$I_{bNS\_X} = P_{bNSC\_X} \cdot GHK_X, \quad X = (K,\ Na) \quad \text{(Eq. S96)}$$

$P_{bNSC\_K} = 0.00014 \cdot C_m, \quad P_{bNSC\_Na} = 0.00035 \cdot C_m$

Calcium-activated background cation current($I_{l(Ca)}$)

$$I_{l(Ca)} = I_{l(Ca)\_Na\_iz} + I_{l(Ca)\_K\_iz} + I_{l(Ca)\_Na\_blk} + I_{l(Ca)\_K\_blk} \quad \text{(Eq. S97)}$$

$$I_{l(Ca)\_X\_a} = P_{l(Ca)\_X} \cdot f_{l(Ca)\_a} \cdot GHK_X \cdot p(O)_a \quad X = (Na,\ K), \quad a = (blk,\ iz) \quad \text{(Eq. S98)}$$

$P_{l(Ca)\_Na} = 0.00273 \cdot C_m$

$P_{l(Ca)\_K} = P_{l(Ca)\_Na}$

$$p(O)_a = \frac{1.0}{1.0+\left(\frac{0.0012}{[Ca^{2+}]_a}\right)^3} \quad \text{(Eq. S99)}$$

Fraction of $I_{l(Ca)}$

$f_{l(Ca)\_iz} = 0.1, \quad f_{l(Ca)\_blk} = 0.9$

ATP-sensitive potassium current(I_{KATP})

$$I_{KATP} = G_{KATP} \cdot (V_m - E_K) \cdot p(O)_{KATP} \cdot x_{IKATP} \quad \text{(Eq. S100)}$$

$G_{KATP} = 17.674 \cdot C_m$

$$p(O)_{KATP} = \frac{0.8}{1.0+\left(\frac{[ATP]_{cyt}}{0.1}\right)^2} \quad \text{(Eq. S101)}$$

$$x_{IKATP} = 0.0236 \cdot ([K^+]_o)^{0.24} \quad \text{(Eq. S102)}$$

Na^+/K^+ pump current(I_{NaK})

The Na^+/K^+ pump model developed by Oka *et al.* (9) on the framework of Smith and Crampin (10) was used after adjusting Amp_{NaK}.

$$I_{NaK} = Amp_{NaK} \cdot V_{cyt\_NaK} \quad \text{(Eq. S103)}$$

$Amp_{NaK} = 25.1779 \cdot C_m$

$$V_{cyt\_NaK} = V_{step1} \quad \text{(Eq. S104)}$$

$$V_{step1} = \alpha_1^+ \cdot P_{1\_6} - \alpha_1^- \cdot P_7 \quad \text{(Eq. S105)}$$

$$V_{step2} = \alpha_2^+ \cdot P_7 - \alpha_2^- \cdot P_{8\_13} \quad \text{(Eq. S106)}$$

$$V_{step3} = \alpha_3^+ \cdot P_{8\_13} - \alpha_3^- \cdot P_{14\_15} \quad \text{(Eq. S107)}$$

$$V_{step4} = \alpha_4^+ \cdot P_{14\_15} - \alpha_4^- \cdot P_{1\_6} \quad \text{(Eq. S108)}$$

$$I_{NaK\_Na} = Stoi_{NaK\_Na} \cdot I_{NaK} \quad Stoi_{NaK\_Na} = 3 \quad \text{(Eq. S109)}$$

$$I_{NaK\_K} = Stoi_{NaK\_K} \cdot I_{NaK} \quad Stoi_{NaK\_K} = -2 \quad \text{(Eq. S110)}$$

The state transition is described by the four-state model.

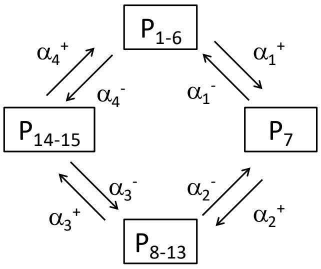

Scheme 3. Na/K pump model

$$\frac{d(P_{1\_6})}{dt} = -\alpha_1^+ \cdot P_{1\_6} + \alpha_1^- \cdot P_7 + \alpha_4^+ \cdot P_{14\_15} - \alpha_4^- \cdot P_{1\_6} \quad \text{(Eq. S111)}$$

$$\frac{d(P_7)}{dt} = -\alpha_2^+ \cdot P_7 + \alpha_2^- \cdot P_{8\_13} + \alpha_1^+ \cdot P_{1\_6} - \alpha_1^- \cdot P_7 \quad \text{(Eq. S112)}$$

$$\frac{d(P_{8\_13})}{dt} = -\alpha_3^+ \cdot P_{8\_13} + \alpha_3^- \cdot P_{14\_15} + \alpha_2^+ \cdot P_7 - \alpha_2^- \cdot P_{8\_13}$$

(Eq. S113)

$$P_{14\_15} = 1 - P_{1_6} - P_7 - P_{8\_13} \quad \text{(Eq. S114)}$$

Rate constants

$$\alpha_1^+ = \frac{k_1^+ \overline{\mathrm{Na}_i}^3}{(1+\overline{\mathrm{Na}_i})^3 + (1+\overline{K_i})^2 - 1} \quad \text{(Eq. S115)}$$

$$\alpha_2^+ = k_2^+$$

$$\alpha_3^+ = \frac{k_3^+ \overline{K_o}^2}{(1+\overline{\mathrm{Na}_o})^3 + (1+\overline{K_o})^2 - 1} \quad \text{(Eq. S116)}$$

$$\alpha_4^+ = \frac{k_4^+ \overline{\mathrm{MgATP}}}{1+\overline{\mathrm{MgATP}}} \quad \text{(Eq. S117)}$$

$$\alpha_1^- = k_1^- [MgATP]_{cyt} \quad \text{(Eq. S118)}$$

$$\alpha_2^- = \frac{k_2^- \overline{\mathrm{Na}_o}^3}{(1+\overline{\mathrm{Na}_o})^3 + (1+\overline{K_o})^2 - 1} \quad \text{(Eq. S119)}$$

$$\alpha_3^- = \frac{k_3^- [Pi][H^+]}{1+\overline{\mathrm{MgATP}}} \quad \text{(Eq. S120)}$$

$$\alpha_4^- = \frac{k_4^- \overline{K_i}^2}{(1+\overline{\mathrm{Na}_i})^3 + (1+\overline{K_i})^2 - 1} \quad \text{(Eq. S121)}$$

$k_1^+ = 0.72,\ k_1^- = 0.08,\ k_2^+ = 0.08,\ k_2^- = 0.008,\ k_3^+ = 4,\ k_3^- = 8000,\ k_4^+ = 0.3,\ k_4^- = 0.2$

$$\overline{\mathrm{Na}_i} = \frac{[\mathrm{Na}^+]_i}{K_{d,Nai}} \quad \text{(Eq. S122)}$$

$$\overline{\mathrm{Na}_o} = \frac{[\mathrm{Na}^+]_o}{K_{d,Nao}} \qquad \text{(Eq. S123)}$$

$$\overline{K_i} = \frac{[K^+]_i}{K_{d,Ki}} \qquad \text{(Eq. S124)}$$

$$\overline{K_o} = \frac{[K^+]_o}{K_{d,Ko}} \qquad \text{(Eq. S125)}$$

$$\overline{\mathrm{MgATP}} = \frac{[MgATP]_{cyt}}{K_{d,MgATP}} \qquad \text{(Eq. S126)}$$

$$K_{d,Nao} = K^0_{d,Nao} \cdot Exp\frac{\Delta_{Nao} \cdot FV_m}{RT} \qquad \text{(Eq. S127)}$$

$$K_{d,Nai} = K^0_{d,Nai} \cdot Exp\frac{\Delta_{Nai} \cdot FV_m}{RT} \qquad \text{(Eq. S128)}$$

$$K_{d,Ko} = K^0_{d,Ko} \cdot Exp\frac{\Delta_{Ko} \cdot FV_m}{RT} \qquad \text{(Eq. S129)}$$

$$K_{d,Ki} = K^0_{d,Ki} \cdot Exp\frac{\Delta_{Ki} \cdot FV_m}{RT} \qquad \text{(Eq. S130)}$$

$K^0_{d,Nai} = 5,\ K^0_{d,Nao} = 26.8,\ K^0_{d,Ki} = 18.8,\ K^0_{d,Ko} = 0.8,\ K_{d,MgATP} = 0.6$

$\Delta_{Nai} = -0.14,\ \Delta_{Nao} = 0.44,\ \Delta_{Ki} = -0.14,\ \Delta_{Ko} = 0.23$

Na+/Ca+ exchange current (I_{NCX})

The NCX model developed by Takeuchi *et al.* (8) was used after adjusting the amplitude factor Amp_{NCX}.

$$\begin{array}{lllll}
E_2Na & \xleftrightarrow{KmNao} & E_2 & \xleftrightarrow{KmCao} & E_2Ca \qquad (1-y) \\
k_1 \uparrow\downarrow k_2 & & & & k_3 \uparrow\downarrow k_4 \qquad \beta \uparrow\downarrow \alpha \\
E_1Na & \xleftrightarrow{KmNai} & E_1 & \xleftrightarrow{KmCai} & E_1Ca \qquad y \\
\alpha_1 \downarrow\uparrow \beta_1 & & \alpha_2 \downarrow\uparrow \beta_2 & & \\
I_1 & & I_2 & &
\end{array}$$

Scheme 4. NCX model

$a = (blk,\ iz)$

$$I_{NCX} = I_{NCX\_iz} + I_{NCX\_blk} \qquad \text{(Eq. S131)}$$

$f_{NCX\_iz} = 0.1, \qquad f_{NCX\_blk} = 0.9$

$$I_{NCX\_Na\_a} = 3 \cdot I_{NCX\_a} \qquad \text{(Eq. S132)}$$

$$I_{NCX\_Ca\_a} = -2 \cdot I_{NCX\_a} \qquad \text{(Eq. S133)}$$

$$I_{NCX\_a} = f_{NCX\_a} \cdot Amp_{NCX} \cdot V_{cyc\_NCX\_a} \qquad \text{(Eq. S134)}$$

$Amp_{NCX} = 61.06 \cdot 0.5 \cdot C_m$

$$V_{cyt\_NCX\_a} = k_1 \cdot q_a(E_1Na) \cdot q(E_1)_{NCX\_a} - k_2 \cdot q_a(E_2Na) \cdot q(E_2)_{NCX\_a} \quad \text{(Eq. S135)}$$

Fraction of I_{NCX}

$f_{NCX\_iz} = 0.1, \quad f_{NCX\_blk} = 0.9$

$$\frac{dp(E_1)_{NCX_a}}{dt} = p(E_2)_{NCX_a} \cdot \alpha_E + p(I_1)_{NCX_a} \cdot \beta_{1_a} + p(I_2)_{NCX_a} \cdot \beta_{2_a} \quad \text{(Eq. S136)}$$

$$-p(E_1)_{NCX_a} \cdot (\beta_{E_a} + \alpha_{1\_X} + \alpha_{2\_X})$$

$$\frac{dp(I_1)_{NCX\_a}}{dt} = p(E_1)_{NCX\_a} \cdot \alpha_{1\_a} - p(I_1)_{NCX\_a} \cdot \beta_{1\_a} \quad \text{(Eq. S137)}$$

$$\frac{dp(I_2)_{NCX\_a}}{dt} = p(E_1)_{NCX\_a} \cdot \alpha_{2\_a} - p(I_2)_{NCX\_a} \cdot \beta_{2\_a} \quad \text{(Eq. S138)}$$

$$p(E_2)_{NCX\_a} = 1 - p(E_1)_{NCX\_a} - p(I_1)_{NCX\_a} - p(I_2)_{NCX\_a} \quad \text{(Eq. S139)}$$

$$q_a(E_1Na) = \frac{1.0}{\left(1.0+\left(\frac{K_{m,Nai}}{[Na^+]_i}\right)^3\right)\cdot\left(1.0+\frac{[Ca^{2+}]_a}{K_{m,Cai}}\right)} \quad \text{(Eq. S140)}$$

$$q_a(E_1Ca) = \frac{1.0}{\left(1.0+\frac{K_{m,Cai}}{[Ca^{2+}]_a}\right)\cdot\left(1.0+\left(\frac{[Na^+]_i}{K_{m,Nai}}\right)^3\right)} \quad \text{(Eq. S141)}$$

$$q(E_2Na) = \frac{1.0}{\left(1.0+\left(\frac{K_{m,Nao}}{[Na^+]_o}\right)^3\right)\cdot\left(1.0+\frac{[Ca^{2+}]_o}{K_{m,Cao}}\right)} \quad \text{(Eq. S142)}$$

$$q(E_2Ca) = \frac{1.0}{\left(1.0+\frac{K_{m,Cao}}{[Ca^{2+}]_a}\right)\cdot\left(1.0+\left(\frac{[Na^+]_o}{K_{m,Nao}}\right)^3\right)} \quad \text{(Eq. S143)}$$

$$\alpha_{1\_a} = q_a(E_1Na) \cdot (f_{Caina\_a} \cdot \alpha_{1\_on} + (1 - f_{Caina\_a}) \cdot \alpha_{1\_off}) \quad \text{(Eq. S144)}$$

$$\beta_{1\_a} = f_{Caina\_a} \cdot \beta_{1\_on} + (1 - f_{Caina\_a}) \cdot \beta_{1\_off} \quad \text{(Eq. S145)}$$

$$\alpha_{2\_a} = f_{Caina\_a} \cdot \alpha_{2\_on} + (1 - f_{Caina\_a}) \cdot \alpha_{2\_off} \quad \text{(Eq. S146)}$$

$$\beta_{2\_a} = f_{Caina\_a} \cdot \beta_{2\_on} + (1 - f_{Caina\_a}) \cdot \beta_{2\_off} \quad \text{(Eq. S147)}$$

$\alpha_{1\_on} = 0.002,\ \alpha_{1\_off} = 0.0015,\ \beta_{1\_on} = 0.0012,\ \beta_{1\_off} = 0.0000005$

$\alpha_{2\_on} = 0.00006,\ \alpha_{2\_off} = 0.02,\ \beta_{2\_on} = 0.18,\ \beta_{2\_off} = 0.0002$

$$\alpha_E = k_2 \cdot q(E_1Na) + k_4 \cdot q(E_2Ca) \quad \text{(Eq. S148)}$$

$$\beta_{E\_a} = k_1 \cdot q_a(E_1Na) + k_3 \cdot q_a(E_2Ca) \quad \text{(Eq. S149)}$$

$$k_1 = exp\left(\frac{0.32 \cdot F \cdot V_m}{R \cdot T}\right) \quad \text{(Eq. S150)}$$

$$k_2 = exp\left(\frac{(0.32-1) \cdot F \cdot V_m}{R \cdot T}\right) \quad \text{(Eq. S151)}$$

$k_3 = 1.0, \quad k_4 = 1.0$

$$f_{Caina\_a} = \frac{[Ca^{2+}]_a}{[Ca^{2+}]_a + K_{m,act}} \quad \text{(Eq. S153)}$$

$K_{m,act} = 0.004$

$K_{m,Nao} = 87.5, \quad K_{m,Nai} = 20.74854, \quad K_{m,Cao} = 1.38, \quad K_{m,Cai} = 0.0184$

Plasma membrane Ca^{2+}-ATPase current (I_{PMCA})

The model equation used in Grandi *et al.* (11) was used for *iz* and *blk* after adjusting the amplitude factor Amp_{PMCA}. H^+ was neglected for convenience.

$$I_{PMCA} = I_{PMCA\_iz} + I_{PMCA\_blk} \quad \text{(Eq. S154)}$$

$$I_{PMCA\_a} = f_{PMCA\_a} \cdot Amp_{PMCA} \cdot \frac{([Ca^{2+}]_a)^{1.6}}{(K_m)^{1.6} + ([Ca^{2+}])^{1.6}} \quad \text{(Eq. S155)}$$

$a = (blk,\ iz)$

$f_{PMCA\_iz} = 0.1,\ f_{PMCA\_blk} = 0.9,\ Amp_{PMCA} = 0.19 \cdot C_m,\ K_m = 0.0005$

CaRU

LCC

The tightly coupled LCC-RyR kinetic model developed by Hinch et al. (12) was used after decreasing P_{RyR} to 5191 from 5967.67 (fL/ms). The new LCC model is described in the section of the L-type Ca^{2+} current.

RyR channel

The state taransition of a RyR is defined by the two-state transition with the activation rate, k_{CO} and deactivation rate k_{OC}. Q_{10} is a temperature factor(Q_{10}=3)

$$k_{co} = Q_{10} \cdot \frac{0.4}{1 + \left(\frac{0.025}{[Ca^{2+}]_{nd}}\right)^{2.7}} \quad \text{(Eq. S156)}$$

The $[Ca^{2+}]_{nd}$ for the activation is,

$[Ca^{2+}]_{nd}$ = Ca_{L0} for LCC-dependent activation of a RyR

$[Ca^{2+}]_{nd}$ = Ca_{00} for spontaneous activation of a RyR

$$k_{oc} = Q_{10} \cdot 0.5664 \quad \text{(Eq. S157)}$$

$$f_t = \frac{k_{co}}{k_{co} + k_{oc}} \quad \text{(Eq. S158)}$$

The state transition of couplon at the regenerative step is also described by the two-state transition scheme.

$$\text{Closed} \underset{k_{roc}}{\overset{k_{rco}}{\rightleftharpoons}} \text{Open}$$

Scheme 5. Two-state model

The activation rate k_{rco} and the deactivation rate k_{roc} are,

$$k_{rco} = f_n \cdot f_t \cdot k_{co} \cdot (sloc0 + [Ca^{2+}]_{SRrl}) \quad \text{(Eq. S159)}$$

$f_n = 7, sloc0 = 0.1$

$[Ca^{2+}]_{nd}$ = Ca_{LR} for LCC-dependent activation,

$[Ca^{2+}]_{nd}$ = Ca_{0R} for RyR-dependent spontaneous activation,

$$k_{roc} = k_{co} \cdot pC^{((N_{RyR}-1)\cdot 0.74)} \quad pC = \frac{k_{oc}}{k_{oc}+f_t \cdot \frac{k_{rco}}{fn}} \quad \text{(Eq. S160)}$$

The f_t is calculated using Ca_{00}. NRyR is the number of RyRs in a couplon and assumed to be 10. The $[Ca^{2+}]_{nd}$ is defined as Ca_{00}, Ca_{0R}, Ca_{L0} or Ca_{LR}.

LCC closed; RyR closed:

$$Ca_{00} = [Ca^{2+}]_{jnc} \quad \text{(Eq. S161)}$$

LCC closed; RyR open:

$$Ca_{0R} = \frac{Ca_{00}+f_R \cdot [Ca^{2+}]_{SRrl}}{1+f_R} \quad \text{(Eq. S162)}$$

$f_R = 0.31$

LCC open; RyR closed:

$$Ca_{L0} = \frac{Ca_{00}+f_L \cdot \frac{\delta V \cdot e^{-\delta V}}{1-e^{-\delta V}}[Ca^{2+}]_o}{\left(1+f_L \cdot \frac{\delta V}{1-e^{-\delta V}}\right)} \quad \text{(Eq. S163)}$$

$f_L = 0.014$

LCC open; RyR open:

$$Ca_{LR} = \frac{Ca_{00}+f_R \cdot [Ca^{2+}]_{SRrl}+f_L \cdot \frac{\delta V \cdot e^{-\delta V}}{1-e^{-\delta V}}[Ca^{2+}]_o}{1+f_R+f_L \cdot \frac{\delta V}{1-e^{-\delta V}}} \quad \text{(Eq. S164)}$$

$$\delta = \frac{2 \cdot F}{R \cdot T} \quad \text{(Eq. S165)}$$

$$\mathrm{p(O)}_t = p(O) + p(O)_{base} \quad \text{(Eq. S166)}$$

$p(O)_{base} = 0.000075$

$$p(O) = Y_{ooo} + Y_{coo} + Y_{cco} + Y_{oco} \quad \text{(Eq. S167)}$$

$$J_{Ca\_rel} = P_{RyR} \cdot \mathrm{p(O)}_t \cdot ([Ca^{2+}]_{SRrl} - [Ca^{2+}]_{jnc}) \cdot Sc\_cell \quad \text{(Eq. S168)}$$

$P_{RyR} = 5191(fL \cdot ms^{-1}) \quad (whole\ cell)$

Sarcoplasmic reticulum Ca^{2+} pump (SERCA) current (J_{SERCA})

The three-state model developed by Trans *et al.* (13) was used after several minor modifications as described Asakura *et al.* (2). The limiting amplitude of J_{SERCA}, amp_{SERCA}, was modified.

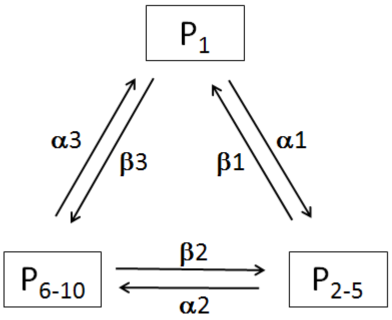

Scheme 6. SERCA model

$$J_{SERCA} = \frac{ampSERCA \cdot V_{cyc}}{2 \cdot F \cdot 1000} \cdot Sc\_cell \qquad \text{(Eq. S169)}$$

$$ampSERCA = 106444.8\ (mmol \cdot ms^{-1})$$

$$V_{cyc} = \frac{6.86 \cdot (\alpha1 \cdot \alpha2 \cdot \alpha3 - \beta1 \cdot \beta2 \cdot \beta3)}{\alpha2 \cdot \alpha3 + \beta1 \cdot \beta2 + \alpha1 \cdot \alpha3 + \beta2 \cdot \alpha1 + \beta2 \cdot \beta3 + \alpha1 \cdot \alpha2 + \beta3 \cdot \beta1 + \beta3 \cdot \alpha2} \qquad \text{(Eq. S170)}$$

$$\alpha1 = 25900 \cdot [MgATP]_{cyt} \qquad \text{(Eq. S171)}$$

$$\alpha2 = \frac{2540}{1 + \left(\frac{Kd_{Cai}}{[Ca^{2+}_{free}]_{blk}}\right)^{1.7}} \qquad \text{(Eq. S172)}$$

$$\alpha3 = \frac{5.35}{1 + \left(\frac{[Ca^{2+}]_{SRup}}{Kd_{Casr}}\right)^{1.7}} \qquad \text{(Eq. S173)}$$

$$\beta1 = \frac{0.1972}{1 + \left(\frac{[Ca^{2+}_{free}]_{blk}}{Kd_{Cai}}\right)^{1.7}} \qquad \text{(Eq. S174)}$$

$$\beta2 = \frac{25435 \cdot [MgADP]_{cyt}}{1 + \left(\frac{Kd_{Casr}}{[Ca^{2+}]_{SRup}}\right)^{1.7}} \qquad \text{(Eq. S175)}$$

$$\beta3 = 149 \cdot [Pi] \qquad \text{(Eq. S176)}$$

$$Kd_{Cai} = 0.0027(mM),\ Kd_{Casr} = 1.378(mM)$$

5. Contraction

The original model Negroni and Lascano (14) was used. Six states of troponin systems (TS), each system composed by 3 adjacent troponin-propomyosin regulatory units acting cooperatively: free TS; Ca^{2+} bound to TS without attached CBs ($TSCa_3$), Ca^{2+} bound to TS with attached CBs in the weak state ($TSCa_{3W}$), Ca^{2+} bound to TS without attached CBs in the power state($TSCa_{3S}$), TS without Ca^{2+} with attached CBs in the power state (TS_S) and TS without Ca^{2+} with attached CBs in the weak state (TS_W).

The rate of ATP hydrolysis by myosin ATPase($ATP_{contraction}$) is calculated by Eq.S177;

$$\frac{dATP_{contraction}}{dt} = \text{ratioATP} \cdot \left(Y_p \cdot [TSCa_w] + Z_q \cdot [TS_w]\right) \cdot V_{cell} \qquad \text{(Eq. S177)}$$

where ratioATP is the stoichiometry of myosin ATPase for each troponin contraction.

Dynamics of the eCB elongation(h)

All equations to calculate the eCB dynamics are the same as in the original model of Negroni and Lascano (14). The half sarcomere length (halfSL) was represented as a sum of the elongation of the equivalent crossbridge (eCB) and the rest X for each of the powered and weakly-bound eCBs. h_p and h_w represent elongation of the elastic component of the strong-bound CB state and weak-bound CB state.

$X_p = halfSL - h_p$ (Eq. S178)

$X_w = halfSL - h_w$ (Eq. S179)

The rate of change in X(dX/dt) is,

$\frac{dX_p}{dt} = B \cdot (h_p - h_{pr})$ (Eq. S180)

$\frac{dX_w}{dt} = B \cdot (h_w - h_{wr})$ (Eq. S181)

Rated parameters are given in Table S7, and A_p and A_w are the stiffness summed for each type eCBs within a cell. The L_e and K_e are the spring constant of the parallel elastic component.

Developed tension

CB force

CB force (F_b) is given by the sum of weak CB force (F_{bw}) and powered CB force (F_{bp}).

$F_b = F_{bw} + F_{bp}$ (Eq. S182)

$F_{bw} = n_{CaBS} \cdot A_w \cdot ([TSCa_{3w}] + [TS_w]) \cdot h_w$ (Eq. S183)

$F_{bp} = n_{CaBS} \cdot A_p \cdot ([TSCa_{3s}] + [TS_s]) \cdot h_p$ (Eq. S184)

The force of the parallel elastic element

$F_p = K_e \cdot (L_{\mathrm{hSL}} - L_0)^5 + L_e \cdot (L_{\mathrm{hSL}} - L_0)$ (Eq. S185)

The detachment rates of CB

$g = Z_a \cdot Y_v \cdot \left(1 - e^{-\gamma \cdot (h_w - h_{wr})^2}\right)$ (Eq. S186)

$g_d = Y_d \cdot Y_c \cdot (L - L_c)^2 + Y_{vd} \cdot \left(1 - e^{-\gamma \cdot (h_w - h_{wr})^2}\right)$ (Eq. S187)

$F_h = \begin{cases} 0.1; h_w > h_{wr} \\ 1; h_w \leq h_{wr} \end{cases}$ (Eq. S188)

Calculation of state transitions of the model

Y_b, Z_b, f, g, Y_p, Z_p, Y_r, Z_r, Y_q, Z_q and g_d are rate constants of the reaction.

$\frac{d[TSCa_3]}{dt} = Y_b \cdot [TS] \cdot \left(\left[Ca_{free}^{2+}\right]_{blk} \cdot 1000\right)^3 - Z_b \cdot [TSCa_3] + g \cdot [TSCa_{3w}] - f \cdot [TSCa_3]eff$ (Eq. S189)

$\frac{d[TSCa_{3w}]}{dt} = f \cdot [TSCa_3]eff - g \cdot [TSCa_{3w}] + Z_p \cdot [TSCa_{3s}] - Y_p \cdot [TSCa_{3w}]$ (Eq. S190)

$$\frac{d[TSCa_{3s}]}{dt} = Y_p \cdot [TSCa_{3w}] - Z_p \cdot [TSCa_{3s}] + Z_r \cdot [TS_s] \cdot \left(\left[Ca^{2+}_{free}\right]_{blk} \cdot 1000\right)^3 - Y_r \cdot [TSCa_{3s}] \quad \text{(Eq. S191)}$$

$$\frac{d[TS_s]}{dt} = Y_r \cdot [TSCa_{3s}] - Z_r \cdot [TS_s] \cdot \left(\left[Ca^{2+}_{free}\right]_{blk} \cdot 1000\right)^3 + Z_q \cdot [TS_w] - Y_q \cdot [TS_s] \quad \text{(Eq. S192)}$$

$$\frac{d[TS_w]}{dt} = Y_q \cdot [TS_s] - Z_q \cdot [TS_w] - g_d \cdot [TS_w] \quad \text{(Eq. S193)}$$

$$[TS] = [TS_{tot}] - [TSCa_3] - [TSCa_{3w}] - [TSCa_{3s}] - [TS_s] - [TS_w] \quad \text{(Eq. S194)}$$

$$[TSCa_3]eff = e^{-R(L-La)^2} \cdot [TSCa_3] \quad \text{(Eq. S195)}$$

Table S7. Parameters in HyBrid model

Parameter	Hybrid model	Units
A_p	2700	$mNmm^{-2}\mu m^{-1}\mu M^{-1}$
A_w	540	$mNmm^{-2}\mu m^{-1}\mu M^{-1}$
B_p	0.5	ms^{-1}
B_w	0.2	ms^{-1}
γ	28000	μm^{-2}
f	0.0023	ms^{-1}
h_{pr}	0.006	μm
h_{wr}	0.0001	μm
K_e	105000	$mNmm^{-2}\mu m^{-5}$
L_a	1.15	μm
L_e	10	$mNmm^{-2}\mu m^{-1}$
$[TS_{tot}]$	23	μM
n_{CaBS}	3	unitless
R	15	μm^{-2}
Y_b	0.1816	$\mu M^{-3}ms^{-1}$
Y_c	1.0	$ms^{-1}\mu m^{-2}$
Y_d	0.0333	ms^{-1}
Y_p	0.1397	ms^{-1}
Y_q	0.2328	ms^{-1}
Y_r	0.1397	ms^{-1}
Y_v	1.5	ms^{-1}
Z_a	0.0023	ms^{-1}
Z_b	0.1397	ms^{-1}
Z_p	0.2095	ms^{-1}

Z_q	0.3724	ms^{-1}
Z_r	7.2626	ms^{-1}

A_p	parameter describing the bridge force of the power work state
A_w	parameter describing the bridge force of the weak state
B	parameter describing equivalent CB kinetics
CB	attached cross-bridge
f	kinetic reaction constant for the CB attaching step
F_b	total CB force
F_bp	CB force of attached CB in power states
F_bw	CB force of attached CB in weak states
F_h	parameter for asymmetry
F_p	parallel force
g	parameter representing CB detachment step from cAM_Dpw
γ	parameter describing Y_v detachment pathway
h_p	mean elongation of attached CBs in power work state
h_w	mean elongation of attached CBs in weak state
h_pr	steady elongation of attached CBs in power work state
h_wr	steady elongation of attached CBs in weak state
K_e	parameter describing the parallel elastic element
L_hSL	half sarcomere length
L_0	parameter describing the parallel elastic element
L_a	parameter constraining the [cM_DP], giving effective [cM_DP]
L_e	parameter describing the parallel elastic element
n_{CaBS}	number $n$$Ca^{2+}$ of CBs for a single TS
R_a	parameter describing the kurtosis of the curve
X_p	non-elastic portion of the contractile element equals to $L_\mathrm{hSL} - h_\mathrm{p}$
X_w	non-elastic portion of the contractile element equals to $L_\mathrm{hSL} - h_\mathrm{w}$

6. Metabolic parameters

Table S8. Values of the model parameters

Parameters	Values	Units
$[Pi_{free}]_{cyt}$	0.50872066859173026	mM
$[AMP]_{cyt}$	0.00033459021041526427	mM
$[ADP_{free}]_{cyt}$	0.0022536111580301241	mM

$[MgADP]_{cyt}$	0.025978226605534577	mM
$[ATP_{free}]_{cyt}$	0.039789862258604494	mM
$[MgATP]_{cyt}$	6.631643709767415	mM
$[Cr]_{cyt}$	12.6772372798697	mM

ATP consumption:

$$v_{cons} = \frac{dATP_{membrane}}{dt} + \frac{dATP_{contraction}}{dt} \quad \text{(Eq. S196)}$$

$$\frac{dATP_{membrane}}{dt} = \frac{dATP_{NaK}+dATP_{PMCA\_blk}+dATP_{PMCA\_iz}+dATP_{SERCA}}{v_{cyt}} \quad \text{(Eq. S197)}$$

7. Initial values of HuVEC model

Vm = -91.4466885079348

Vo = 0

Vi = Vm + Vo

TnChCa = 0.110742559707052 * 0.9

CaMCa = 0.000228581865602447 * 0.9

bufferSRCa = 0.00172960014640511 * 0.9

Lb_jnc = 0.0218215322629436

Lb_iz = 0.0075621764602356

Hb_jnc = 0.185094540066232

Hb_iz = 0.0769149150028914

Nai = 6.66894310282034

Ki = 139.238265011042

Catot_jnc = 0.207176351449979

Catot_iz = 0.084640522722006

Catot_blk = 0.11279654524634

Ca_SRup = 0.761077662687456

Catot_SRrl = 2.21876221622152

O_TM = 0.000000706725155695262

I2_TM = 0.0117704053067285

Is_TM = 0.304002781414015

```
Is_LSM = 0.2
I2_LSM = 0
C_LSM = 1.0 - Is_LSM - I2_LSM
fixzero_INaLslowgate = 0

Yco_iz = 0.992251726297519
Yoc_iz = 0.000000024556270151713
Yoo_iz = 0.00000314564543512061
Yco_blk = 0.992424981547859
Yoc_blk = 0.0000000240070147854924
Yoo_blk = 0.00000314619469048683

Yooo = 0.00000172489315884865
Yooc = 0.00000142034754677507
Ycoo = 0.0000138422676498755
Ycoc = 0.992110534408681
Ycco = 0.0000000953816272498217
Yoco = 0.000000000000156949238162028
Yocc = 0.0000000249594301562175

y1_IKr = 0.0000440909425988806
y2_IKr = 0.000003386
y3_IKr = 0.999363731

paraxs1_iz = 0.277482694590328
paraxs2_iz = 0.000131110342877451
paraxs1_blk = 0.277482694590328
paraxs2_blk = 0.000131110342877451

Ov_IKs = 0.01
C2IKs_iz = 0.2
OcIKs_iz = 0.01
C2IKs_blk = 0.2
OcIKs_blk = 0.01

a_IKto = 0.000793627635934239
```

y1_IKto = 0.999756080468878
y2_IKto = 0.575995954010486

Pbspm = 0.594875991179992

E1NCX_iz = 0.238718640001014
I1NCX_iz = 0.13771129457898
I2NCX_iz = 0.622892868847556
E1NCX_blk = 0.111872123711613
I1NCX_blk = 0.203023555446362
I2NCX_blk = 0.684869019924837

P1_6_NaK = 0.435289193632868
P7_NaK = 0.0831770174499825
P8_13_NaK = 0.281082409575779

halfSL = 1.09840500012898
Fb = 0.0502092089156129
Fp = 4.94926096641491
TSCa3 = 0.00899891910620064
TSCa3W = 0.000369547640656701
TSCa3S = 0.000153834503967436
TSS = 0.000876347322180234
TSW = 0.000492054058977473
hw = 0.000100147615113241
hp = 0.00600014761511324

ATPt_cyt = 6.67701543987464
ADPt_cyt = 0.0227671477707
Pi_cyt = 0.381130087573153
PCr_cyt = 13.9261301893242

ATPt_mit = 6.20328024045349
Pi_mit = 2.23625256468335
NADH_mit = 1.60566548407208
H_mit = 0.0000285579652594013

K_mit = 117.80018539511

UQr_mit = 0.201994111666095

ctCrd_mit = 0.0448941156153114

dpsi = -159.077387598919

8. References

1. Shannon, T.R.; Wang, F.; Puglisi, J.; Weber, C.; Bers, D.M. A mathematical treatment of integrated Ca dynamics within the ventricular myocyte. *Biophys. J.* **2004**, *87*, 3351–3371.
2. Negroni, J.A.; Lascano, E.C. Simulation of steady state and transient cardiac muscle response experiments with a Huxley-based contraction model. *J. Mol. Cell. Cardiol.* **2008**, *45*, 300–312.
3. Rush, S.; Larsen, H. A practical algorithm for solving dynamic membrane equations. *IEEE Trans. Biomed. Eng.* **1978**, *25*, 389–392.
4. Ishihara, K.; Yan, D.H. Low-affinity spermine block mediating outward currents through Kir2.1 and Kir2.2 inward rectifier potassium channels. *J. Physiol.* **2007**, *583*, 891–908.
5. Takeuchi, A.; Tatsumi, S.; Sara, N.; Terashima, K.; Matsuoka, S.; Noma, A. Ionic mechanisms of cardiac cell swelling induced by blocking Na^+/K^+ pump as revealed by experiments and simulation. *J. Gen. Physiol.* **2006**, *128*, 495–507.
6. Oka, C.; Cha, C.Y.; Noma, A. Characterization of the cardiac Na^+/K^+ pump by development of a comprehensive and mechanistic model. *J. Theor. Biol.* **2010**, *265*, 68–77.
7. Smith, N.P.; Crampin, E.J. Development of models of active ion transport for whole-cell modelling: cardiac sodium-potassium pump as a case study. *Prog. Biophys. Mol. Biol.* **2004**, *85*, 387–405.
8. Grandi, E.; Pasqualini, F.S.; Pes, C.; Corsi, C.; Zaza, A.; Severi, S. Theoretical investigation of action potential duration dependence on extracellular Ca2+ in human cardiomyocytes. *J. Mol. Cell. Cardiol.* **2009**, *46*, 332–342.
9. Hinch, R.; Greenstein, J.L.; Tanskanen, A.J.; Xu, L.; Winslow, R.L. A simplified local control model of calcium-induced calcium release in cardiac ventricular myocytes. *Biophys. J.* **2004**, *87*, 3723–3736.
10. Tran, K.; Smith, N.P.; Loiselle, D.S.; Crampin, E.J. A thermodynamic model of the cardiac sarcoplasmic/endoplasmic Ca^{2+} (SERCA) pump. *Biophys. J.* **2009**, *96*, 2029–2042.
